# The effects of waiting time for outpatient psychotherapeutic interventions on patient-reported outcomes in adolescents and adults with eating disorders: a systematic review and meta-analysis

**DOI:** 10.1186/s40337-026-01660-4

**Published:** 2026-06-05

**Authors:** Marike Kranes, Pauline S. Münchenberg, Carmen Thomas, Ricarda S. Schulz, Alessandro Campione, Anika Kreutzberg

**Affiliations:** 1 Charité – Universitätsmedizin Berlin, Corporate Member of Freie Universität Berlin and Humboldt-Universität zu Berlin, Berlin, Germany; 2 Institute of Public Health, Charité – Universitätsmedizin Berlin, Corporate Member of Freie Universität Berlin and Humboldt-Universität zu Berlin, Berlin, Germany; 3 Department of Health Care Management, Technische Universität Berlin, Berlin, Germany

**Keywords:** Anorexia nervosa, Bulimia nervosa, Binge-eating disorder, Other specified feeding or eating disorder, Outpatient psychotherapy, Access, Waiting time, Waitlist control groups, Treatment delay, Patient-reported outcomes

## Abstract

**Objective:**

To assess the effects of waiting time for outpatient psychotherapeutic interventions on patient-reported outcomes in adolescents and adults with eating disorders.

**Method:**

MEDLINE, Embase, APA PsycInfo, CENTRAL and BASE were searched on 12 August 2025. Quantitative studies of adolescents (≥ 10 years) and adults with eating disorders that examined waitlist conditions prior to outpatient psychotherapeutic interventions and measured eating disorder pathology with the Children's Eating Attitudes Test (chEAT) or Eating Disorder Examination-Questionnaire (EDE-Q) were eligible. Risk of bias was assessed using the RoB 2 and ROBINS-I tools. A within-group random-effects meta-analysis was performed assessing pre–post change scores of the EDE-Q Global Score (primary analysis) and EDE-Q subscales (secondary analyses). A full protocol has been pre-registered on PROSPERO (registration number: CRD420251090691).

**Results:**

Nine waitlist-controlled studies (total n = 647, waitlisted n = 283) including adult participants with bulimia nervosa, binge-eating disorder and 'eating disorder not otherwise specified' met our eligibility criteria. Eight studies (waitlisted n = 273) were included in the meta-analysis. Participants entered the waiting period with high EDE-Q Global Scores, ranging from 3.08 to 4.65 across studies. The pooled pre–post effect estimate was Hedges’ g = − 0.17 (95% CI − 0.30 to − 0.05), indicating a negligible reduction in eating disorder pathology. Certainty of evidence was rated as moderate, given the high risk of bias across studies.

**Discussion:**

Based on our results, waiting in waitlist control groups represents a period during which substantial eating disorder pathology and associated distress persist without therapeutic intervention. Further research is needed to better understand the effects of waiting time for regular eating disorder treatment outside a study setting.

**Supplementary Information:**

The online version contains supplementary material available at 10.1186/s40337-026-01660-4.

## Abstract (plain language summary)

Study participants on a waitlist to be treated for bulimia nervosa, binge-eating disorder, and ‘eating disorder not otherwise specified’ continued to report high levels of eating disorder symptoms while waiting about 10 weeks for psychotherapy. This means that individuals may experience considerable distress during a delay in starting treatment. However, being on a study waitlist is not the same as waiting for treatment in everyday healthcare. In a study, participants know when and what kind of treatment they will get, which might already help reduce symptoms before treatment even starts. We could not find studies that assessed the effects of waiting time in everyday healthcare.

**Supplementary Information:**

The online version contains supplementary material available at 10.1186/s40337-026-01660-4.

## Introduction

### Background

Timely access to outpatient psychotherapy is considered a critical factor in the effective treatment of eating disorders (EDs) [1]. EDs are a heterogeneous group of mental health conditions, characterised by persistently disturbed eating or eating-related behaviour that results in altered food intake and significantly impairs the physical health or psychosocial functioning of affected individuals [2]. Within the feeding and eating disorder category, the Diagnostic and Statistical Manual of Mental Disorders, Fifth Edition (DSM-5) [2] defines, among other disorders, three EDs that are mutually exclusive within a given episode: binge-eating disorder (BED), bulimia nervosa (BN), and anorexia nervosa (AN), with prevalence typically reported as highest for BED and lowest for AN. While research is mainly conducted on these more recognised clinical presentations, the highest prevalence among EDs is found in 'other specified feeding or eating disorder' (OSFED) [3]. OSFED is diagnosed in cases where feeding or eating related symptoms cause clinically significant distress or impairment in key areas of functioning but do not meet the full diagnostic criteria of any of the specific disorders in the category. This includes but is not limited to atypical AN (AAN), BN of low frequency and/or limited duration (sub-BN), BED of low frequency and/or limited duration (sub-BED), purging disorder (PD), and night eating syndrome (NES) [2].

The burden of EDs is increasing, especially in young people. A recent review by Silén and Keski-Rahkonen [4] reports that 5.5–17.9% of young females and 0.6–2.4% of young males have experienced an ED by early adulthood. Early and effective treatment is crucial to reduce the duration of untreated disease and alter its deteriorating course [5], often characterised by persistence, relapses, and transitions between diagnoses [6–10]. This is further emphasised by the significantly elevated mortality rates among affected individuals with a standardised mortality ratio (SMR) of 3.39, and particularly those with AN, with a SMR of 5.21 [11].

The medical complications and comorbidities that arise in or cooccur with EDs are manifold and can affect every organ system [12, 13]. Even after recovery, EDs are associated with adverse long-term health outcomes [14, 15]. However, the elevated mortality arises not only from the physical consequences but also from increased suicidality [16, 17]. Alongside EDs, psychiatric comorbidities are highly prevalent, with approximately 70% of individuals being also affected by at least one other former Axis I disorder [8, 18, 19]. The most common comorbid disorders are anxiety and mood disorders, substance use disorders (SUDs), post-traumatic stress disorders and, on former Axis II, personality disorders [20, 21].

Although treatment recommendations vary between disorders and across guidelines, psychotherapy consistently plays a central role in ED management [22]. However, due to increasing demand and limited mental health service capacity, long waiting times are common in many healthcare systems [23]. Delays in starting psychotherapeutic treatment may lead to individuals dropping out while waiting [24, 25], worsening symptoms or adverse long-term outcomes such as chronicity [26]. These unmet needs not only have (socio-)economic consequences [27, 28] but primarily affect patients and their quality of life (QoL) [29–32]. Although the occurrence of long waiting times is well recognised, there is still a lack of evidence regarding the impact of these delays on patient-reported outcomes (PROs). PROs shift the focus towards the patient's perspective when assessing health outcomes. This enables a re-evaluation of healthcare services, ultimately improving treatment quality and value [33–36]. To promote standardised, patient-centred outcome assessment, international experts from professional and lived-experience backgrounds developed a consensus-based set of outcomes under the coordination of the International Consortium for Health Outcomes Measurement (ICHOM) [33].

In the context of EDs, this patient-centred perspective is highly relevant when considering the DSM-5 severity specifiers, which are based on body mass index (BMI) (AN), frequency of inappropriate compensatory behaviours per week (BN), and frequency of binge-eating episodes per week (BED). Their validity as proxies for clinical severity has been questioned and previous studies demonstrated only limited support [37–40] or even found reverse effects [41, 42]. Although weight stabilisation may be clinically necessary, particularly in individuals who are critically underweight, BMI normalisation does not automatically correspond to reductions in ED cognitions or psychological distress [43, 44].

### Current state of research and objective

Cognitive behavioural therapy (CBT) is often recommended as a first-line psychological treatment for EDs [45–47] and has shown to be efficacious in several meta-analyses, outperforming waitlist controls in BN, BED [48–51] and OSFED [48]. Linardon et al. [50] found superiority of CBT over other treatments in BN and BED, but not in AN, which is aligned with the results of subsequent meta-analyses [52–54]. In contrast, Bruns et al. [48] could not find superiority of CBT for any of these diagnoses, additionally including OSFED.

Therefore, other validated treatment modalities should also be considered. Furthermore, there is a growing body of evidence on newer interventions, including internet-based treatment and guided self-help (GSH) [55, 56]. This is particularly important in light of limited service capacity [57], administrative hurdles [58], poor service utilisation [59], and insufficient treatment acceptance [60], which necessitate low-threshold interventions [61]. Previous research has extensively examined these barriers and why many individuals with EDs do not seek or decline treatment [62–64]. Austin et al. [65] systematically synthesised the available evidence on the duration of untreated ED (DUED) in help-seeking individuals. Their review included an examination of cross-sectional associations between DUED and both symptom severity and long-term outcomes, operationalised respectively as BMI and the presence of a persisting ED diagnosis after treatment. The findings suggest that DUED may be a modifiable factor influencing ED outcomes. Less attention has been paid to the effects of waiting in individuals who actively seek help but are unable to access care, which highlights the gap in knowledge regarding treatment delays.

Analyses of routine or survey data on waiting time and its effects are scarce. Reichert and Jacobs [66] evaluated routine data from early intervention in psychosis services in England and found that longer waiting times were significantly associated with a deterioration of PROs. Similarly, in a retrospective analysis of routine outpatient data from the Netherlands, longer waiting times were significantly associated with poorer treatment outcomes in individuals with major depressive disorder [67]. Due to the limited existence of such studies, randomised controlled trials (RCTs) using waitlist control groups (WLCGs) can be an additional source when assessing the effects of waiting time. Steinert et al. [68] and Penkova et al. [69] utilised these WLCGs in the context of social anxiety disorder and panic disorder to conduct meta-analyses and found only small symptom changes over short-term waiting periods of approximately 10 weeks, indicating negligible symptom improvements in the absence of treatment. It remains unclear if and how different service-related waiting times impact PROs in adolescents and adults with EDs.

While previous research has examined broader constructs such as the DUED, this concept encompasses delays occurring both prior to help-seeking and within the healthcare system, thereby limiting insight into the specific effects of service-related delays after initial contact with care. In particular, little is known about the impact of the waiting period between help-seeking and the initiation of outpatient psychotherapeutic treatment, during which individuals have already entered the healthcare system but remain without active intervention. Furthermore, existing evidence has predominantly focused on clinical or diagnostic outcomes, with comparatively little attention to PROs that capture symptom burden and distress from the patient's perspective.

Synthesised evidence is needed to understand the consequences of treatment delays from a patient-centred perspective and to inform mental healthcare practice, as well as health policymakers. Therefore, the aim of this systematic review and meta-analysis was to assess the effects of waiting time for outpatient psychotherapeutic interventions on PROs in adolescents and adults with AN, BN, BED, and OSFED.

## Methods

### Research question and protocol registration

We conducted a systematic review and meta-analysis following the Cochrane Handbook for Systematic Reviews of Interventions [70]. The research question 'What are the effects of waiting time for outpatient psychotherapeutic interventions on PROs in adolescents and adults with AN, BN, BED and OSFED' was formulated using the Population, Exposure, Comparator, Outcome (PECO) framework (Additional file 1). Reporting adheres to the Preferred Reporting Items for Systematic reviews and Meta-Analyses (PRISMA) 2020 guideline [71]. The completed checklist with locations of item reporting is provided in Additional file 2.

A full protocol has been pre-registered on PROSPERO (registration number: CRD420251090691). All subsequent amendments to the protocol were documented and can be accessed through the PROSPERO version history.

### Eligibility criteria

Studies were *included* if they

1. used an empirical, quantitative design, either randomised or non-randomised, such as RCTs, clinical trials, quasi-experimental studies or cohort studies;
2. involved adolescents (≥ 10 years, according to the World Health Organization [72] definition) and/or adults (≥ 18 years) with AN, BN, BED or OSFED, according to DSM or International Statistical Classification of Diseases and Related Health Problems (ICD) (research) criteria that were valid at the time of diagnosis, or who exceeded validated cut-off values in questionnaires or interviews based on DSM or ICD (research) criteria for AN, BN, BED or OSFED that are explicitly stated to be used as a proxy for full diagnosis. In addition to the example diagnoses listed under OSFED in the DSM-5, i.e. AAN, sub-BN, sub-BED, PD, and NES, other EDs diagnosed as OSFED were also eligible. Sub-BN and sub-BED had to be explicitly classified as OSFED for eligibility, or their diagnostic criteria had to be described in accordance with the DSM or ICD criteria for OSFED;
3. either (a) compared an active psychotherapeutic intervention group (IG) in an outpatient setting with a WLCG or a no-treatment control group, or (b) retrospectively analysed a cohort of patients who ultimately received outpatient psychotherapy. An active psychotherapeutic intervention was defined as any recognised form of psychotherapy or any psychotherapeutically-based intervention, delivered individually or in groups, either in-person, via real-time online sessions, phone calls, messages, or in digital formats (e.g. via an app or online course). During the intervention, contact with a mental health professional or mental health trainee (postgraduate) had to be provided at least once after the initial consultation, diagnostic workup or study enrolment;
4. included a waiting period ≥ 4 weeks for the WLCG, no-treatment group or cohort of patients who ultimately received outpatient psychotherapy;
5. reported ED-specific PROs, measured using the Children's Eating Attitudes Test (chEAT) [73], Eating Disorder Examination-Questionnaire (EDE-Q) [74] or EDE-Q 6.0 [75], for all study groups separately; and
6. reported these PROs for a minimum of two time points: (1) at or near the time of initial consultation, diagnostic workup, or study enrolment (baseline), and (2) at least once at a subsequent time point (i.e. at intervention start and/or at follow-up).

Due to equipoise concerns regarding the randomisation to WLCGs when examining validated treatments (e.g. CBT), it was not justifiable to only include RCTs. Therefore, we also searched for non-randomised studies of interventions (NRSIs), such as cohort studies, to get further insights into the natural clinical course of affected individuals while waiting for treatment. Studies that included only a subset of participants meeting the age cut-off were only eligible if most participants were aged 10 years or older, or if data were reported separately for the age groups considered.

As OSFED was only introduced in the DSM-5 [2] and ICD-11 [76], the former diagnosis 'eating disorder not otherwise specified' (EDNOS), introduced in the DSM-III-R [77], was considered equivalent. BED was also only introduced in the DSM-5 and ICD-11. Prior to that, BED had to be diagnosed as EDNOS or using the BED research criteria, introduced in the DSM-IV [78] as criteria set for further study.

The ≥ 4 weeks cut-off for the waiting period of WLCGs was chosen based on the EDE-Q reference period of 28 days. The EDE-Q is a self-report version of the semi-structured Eating Disorder Examination (EDE) interview [79] and assesses eating pathology through 22 items on a seven-point rating scale across the subscales Restraint, Weight Concern, Shape Concern, and Eating Concern. Results are reported as mean scores with standard deviations (SDs). A Global Score can be calculated as the mean of the four subscale scores. Scores range from 0 to 6, with higher values indicating greater pathology. Additionally, six items assess the frequency of ED core behaviours, such as binge eating and purging [74]. The current version, the EDE-Q 6.0, contains only minimal linguistic adaptations [75]. The 22 items contributing to the Global Score are identical, making the versions highly comparable [80]. Extensive research has demonstrated good overall internal consistency and test–retest reliability across ED diagnoses [81–84].

Generic PROs of well-being or functioning (measured using the KIDSCREEN-10 [85] or the WHO Disability Assessment Schedule 2.0 [86]), other mental health-related PROs, i.e. measures of anxiety, depression, and suicidal ideation (measured using the Revised Children's Anxiety and Depression Scale-25 [87], Patient Health Questionnaire-2 [88], Patient Health Questionnaire-9 [89], Generalised Anxiety Disorder-2 or Generalised Anxiety Disorder-7 [90]), as well as ED-specific QoL and social functioning (measured using the Clinical Impairment Assessment [91]) were eligible as secondary outcomes, but not required for inclusion.

During the work process, the eligibility criteria have been amended once to ensure precision, clarity, and reproducibility. Additionally, the eligible patient-reported outcome measures (PROMs) have been specified to enhance comparability between the individual studies. This specification was made in accordance with the ICHOM recommendations [33] to follow international consensus and ensure participation of persons with lived experience in the decision-making process. The amendments were made prior to data extraction and have been documented in version 2.0 of the registration record.

Studies were *excluded* if they

1. did not use an empirical, quantitative design, e.g. case reports, case series, conference abstracts, editorials, and commentaries. Additionally, other reviews and meta-analyses were excluded;
2. involved participants with.
  - disorders diagnosed as OSFED that are feeding-related and/or whose occurrence in adolescence or adulthood is associated with neurodevelopmental disorders,
  - avoidant/restrictive food intake disorder (ARFID), pica, rumination disorder (RD) or an ‘unspecified feeding or eating disorder’ (UFED),
  - bipolar disorders, psychotic disorders, or active SUDs, except for nicotine, and did not report results separately for participants with AN, BN, BED or OSFED without these diagnoses

(3) explicitly stated permission for participants to undergo other active psychotherapeutic interventions for their ED or other mental disorder(s) during the study period;
(4) used control groups that receive any kind of intervention, such as treatment as usual or minimal intervention; or.
(5) examined.
  - caregiver, family or spousal interventions,
  - active psychotherapeutic interventions in an outpatient setting prior to inpatient treatment, with the explicit objective of bridging the waiting period until hospital admission,
  - active psychotherapeutic interventions in an outpatient setting immediately following inpatient treatment (relapse prevention),
  - unguided self-help or other interventions that do not include contact with a mental health professional or mental health trainee (postgraduate),
  - pharmacotherapeutic treatment without an active psychotherapeutic intervention, or.
  - other forms of therapy that are not psychotherapeutic in approach, such as movement, art or nutrition therapy.

Rationales for the exclusion criteria are provided in Additional file 3.

### Study identification and selection process

MEDLINE (via Ovid; from 1946), Embase (via Ovid; from 1947), APA PsycInfo (via EBSCO; from 1887), CENTRAL and BASE were searched. The final search was executed on 12 August 2025 across all search engines. The search strategy was developed in Ovid MEDLINE by MK and piloted by searching seed papers [92–95]. It underwent peer review using the Peer Review of Electronic Search Strategies (PRESS) checklist [96], as well as review by an information specialist and a librarian. The review process involved checking included concepts and their respective subject indexing terms, keywords, and synonyms, as well as proofreading of syntax and spelling. Studies were included regardless of publication status. Both peer-reviewed and grey literature were considered. No restrictions were applied regarding publication year or language. The final search strings for all engines can be found in Additional file 4. Search lines that did not yield additional results at the time of the search were retained to ensure conceptual and methodological completeness, particularly for potential future updates of the review.

To identify any additional eligible studies, reference list checking of existing reviews and meta-analyses was performed manually, and citation tracking, i.e. forward and backward citation searching, of the included studies was executed by MK using ResearchRabbit [97] on 9 October 2025. Deduplication of these records and the search engine exports was performed in R (version 4.5.1) [98] using the *revtools* package (version 0.4.1) [99].

The Evidence Review Accelerator (TERA) [100] was used to manage study screening. Screening was carried out independently by MK and either PSM or CT. Any disagreements were resolved through discussion or, if consensus could not be reached, through consultation with AK. Reports written in languages other than English or German, i.e. Persian, Arabic and Dutch, were translated into English by native speakers.

### Data collection and risk of bias assessment

MK and PSM independently extracted data, and MK and CT independently assessed risk of bias. Again, any discrepancies were resolved through discussion or consultation with AK. A standardised data extraction form was developed and piloted on a small number of studies to ensure consistency across studies and between reviewers. Extracted data items are displayed in Additional file 5. Additionally, funding sources and conflicts of interest were reviewed. Corresponding study authors were contacted if WLCG mean values or SDs of the EDE-Q Global Score or subscales for the two mandatory time points were missing. For Schlup et al. [101], we received EDE-Q Global Score and subscale means with SDs for both time points from the authors. Data for Krohmer et al. [102] was extracted from the supplemental material [103]. For Arcelus et al. [104], post-waiting means and post-waiting SDs were unavailable and could not be obtained by contacting the corresponding author. Post-waiting means were derived by adding the reported mean difference (MD) to the pre-waiting mean. As proposed by Morris [105] and Morris and DeShon [106], SDs in pre- and post-test populations can be assumed equal. However, to increase the precision we approximated post-waiting SDs by multiplying the pre-waiting SD by the average proportional change in SDs from pre to post observed in the remaining included studies. ED core behaviours assessed with the EDE-Q, reported as means with SDs, were only extracted if Global Scores and subscales were not reported and could not be obtained.

For risk of bias assessment at the results level, the Cochrane Risk of Bias 2 (RoB 2) tool [107] was used for RCTs, and the Risk Of Bias In Non-randomised Studies of Interventions (ROBINS-I) tool [108] for NRSIs. Given the reversed logic of our objective, we adapted these tools by considering the control condition, i.e. the waitlist, as the intervention. Overall judgement for each study followed the respective algorithms. To visualise the risk of bias results, traffic light plots were generated.

### Data synthesis

Results were displayed in outcome tables, synthesised narratively and summarised in a 'Summary of Findings' table. Due to the expected clinical and methodological diversity across studies, initially no meta-analysis of effect estimates was planned. Therefore, the synthesis was intended to follow the Synthesis Without Meta-analysis (SWiM) reporting guideline [109]. However, following the previous amendment of PROMs, a meta-analysis of effect estimates was possible, as studies were deemed to be sufficiently similar.

### Statistical analyses

We conducted a within-group meta-analysis assessing pre–post change scores of the EDE-Q Global Score (primary analysis) and the EDE-Q subscales (secondary analyses). All statistical analyses were performed in *R* (version 4.5.2) [98] using the *metafor* package (version 4.8.0) [110]. The code for the meta-analysis was written by RSS and independently reviewed by MK and AC.

Assuming that the included studies estimated different but related effects of waiting time, we used random-effects models for all analyses to estimate the mean of the underlying distribution of effects and to account for unexplained heterogeneity [111–113]. Following current best-practice recommendations [113, 114], between-study variance was estimated using a restricted maximum likelihood method [115]. An inverse variance weighting scheme was applied. For all studies, Cohen's d [116] was calculated using the SD within. A pre–post correlation coefficient of r = 0.5 was assumed, consistent with prior methodological findings [117, 118] and similar meta-analyses [68, 69]. Effects of waiting time were analysed using Hedges’ g to correct for small sample sizes [119], with 95% confidence intervals (CIs) calculated using the Wald method [120]. Negative values indicate symptom improvement, whereas positive values indicate symptom deterioration. Forest plots were generated to present results. Statistical heterogeneity was assessed using τ^2^ and I^2^ statistics [121]. For the interpretation of I^2^, the proposed thresholds by Deeks et al. [113] were applied. The ability to detect true between-study heterogeneity was limited due to the within-group design, the use of a single outcome measure and the small number of studies. Thus, the estimated magnitude of heterogeneity was interpreted with caution. For the primary analysis of the Global Score, multiple subgroup and sensitivity analyses were performed.

#### Subgroup analyses

To explore potential sources of heterogeneity, subgroup analyses were conducted for the moderators waiting time (categorised as < 10 weeks versus ≥ 10 weeks) and diagnostic composition (BED versus multiple diagnoses). We did not conduct a meta-regression for waiting time lengths because of the insufficient number of observations (k ≤ 10) per level [113].

#### Sensitivity analyses

**Fig. 1:**
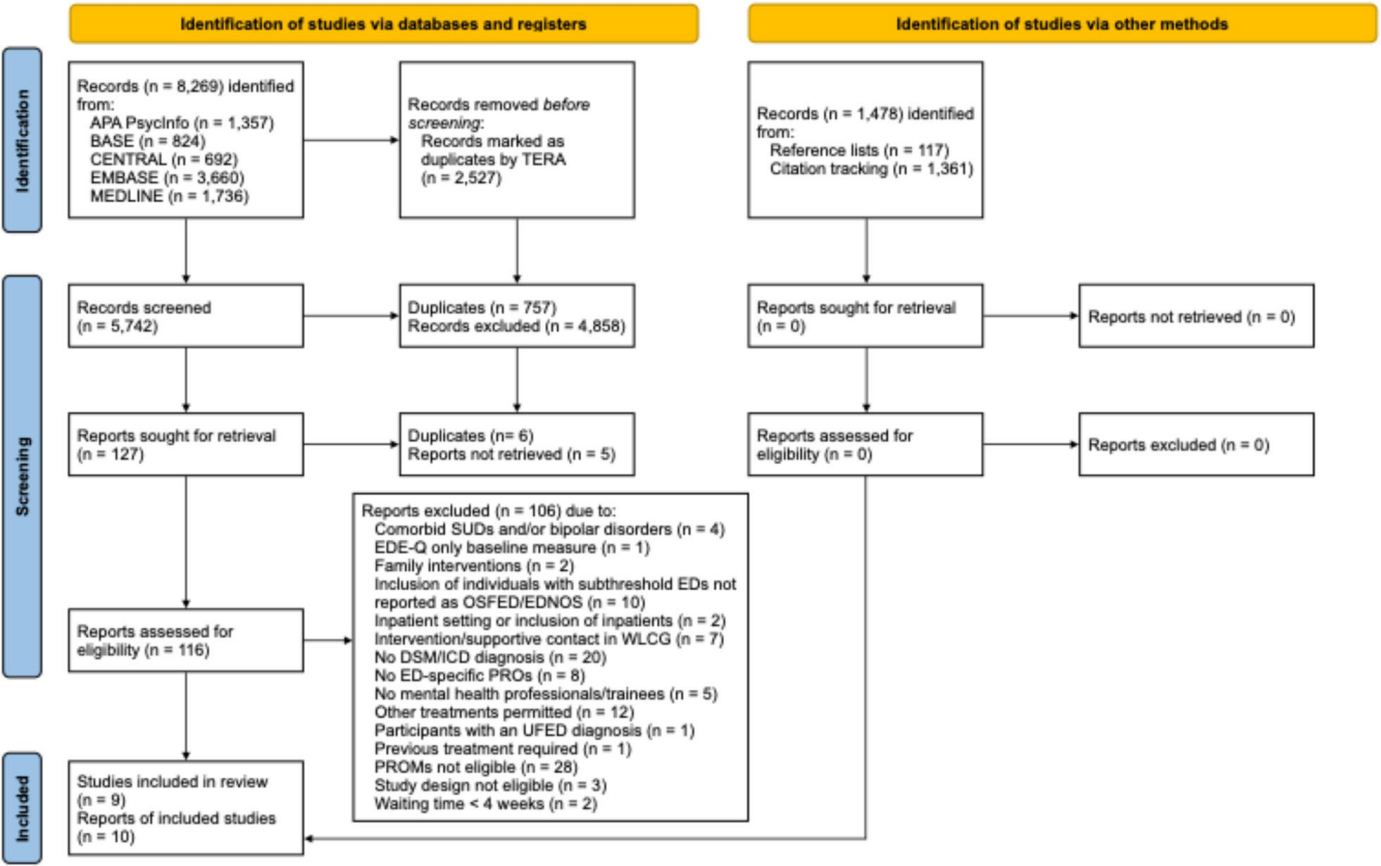
PRISMA 2020 flow diagram. Figure adapted from Page et al. [71]

**Fig. 2:**
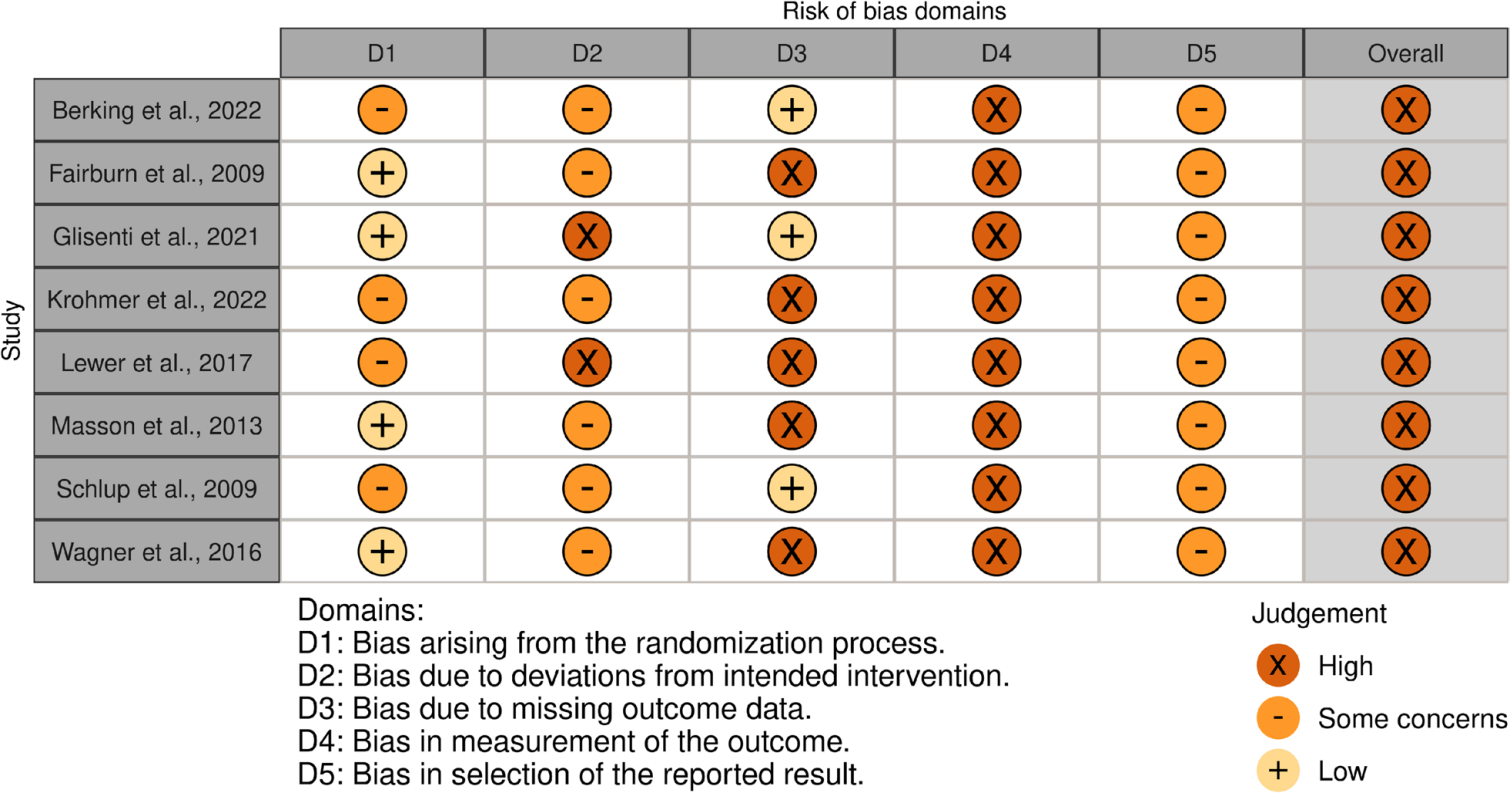
Risk of bias in randomised trials assessed with RoB 2. Traffic light plot generated using robvis [142]

Sensitivity analyses were performed using alternative pre–post correlation coefficients of r = 0.2 and r = 0.8 to examine the robustness of our findings. The upper value was determined via specification analysis, conducted by AC, by plotting the (1) sample-size (Additional file 6**, **Fig. 1) and (2) inverse variance weighted means (Additional file 6**, **Fig. 2), and CIs of Hedges’ g along the grid of correlation coefficients (i.e. r ∈ [-1,1]) across studies. Visual inspection of both plots showed an inflection point in the weighted estimates around r = 0.8, marking the first pronounced change in effect magnitude and precision. For methodological completeness, r = 0.2 was chosen as a symmetrical lower-bound value but was deemed rather unrealistic based on methodological findings that suggest pre–post correlations are usually ≥ 0.5 [117, 118]. This was also supported by the specification plots.

As Arcelus et al. [104] was the only NRSI, and risk of bias was rated as critical, this study was excluded in a further sensitivity analysis to assess its influence on the pooled effect estimates. Krohmer et al. [102] and Lewer et al. [122] adopted measures including an exposure component during baseline assessment. While these tasks were not designed as interventions, they confronted participants with their body and body perception, which might elicit anxiety or discomfort in individuals with EDs. Following a CBT approach, an anxiety-reducing effect could not be ruled out. Krohmer et al. [102] were therefore excluded in an additional sensitivity analysis. Lewer et al. [122] did not report Global Scores.

### Risk of publication bias

Potential publication bias was explored by visual inspection of funnel plots for analyses including ≥ 5 studies. Findings were interpreted with caution due to the within-group design using WLCGs. The estimated effects represent pre–post changes within control conditions rather than between-group treatment effects, for which funnel plot methods were originally developed [123]. Formal statistical tests for funnel plot asymmetry (e.g. Egger’s test) were not performed because of the small number of included studies (k ≤ 10) [124]. Reporting and publication bias were additionally assessed within the Grading of Recommendations, Assessment, Development and Evaluation (GRADE) assessment.

### Certainty of evidence and critical appraisal

The GRADE approach [125] was used to assess the certainty of evidence for each EDE-Q outcome. The assessment was executed by MK and reviewed by PSM.

The A MeaSurement Tool to Assess systematic Reviews (AMSTAR) 2 tool was used to critically appraise the methodological quality of our review upon its completion [126]. Decisions on the overall judgement followed the respective algorithms.

## Results

### Study selection

Of the 8,269 records identified by the search engines, 2,527 were automatically marked as duplicates by TERA, which has been manually checked for and confirmed by MK (Fig. 1). During title/abstract screening, another 757 records were identified as duplicates by MK and PSM. A further 1,478 reports were identified by reference list checking and citation tracking. Of the 127 reports sought for retrieval, six were further duplicates and five could not be retrieved, resulting in 116 full texts assessed for eligibility. Nine studies (ten reports) were finally included. One report was a doctoral dissertation [127]. Data were only extracted from the corresponding peer-reviewed journal publication. Explanations for the exclusion of studies that initially appeared to be eligible are provided in Additional file 7.

### Study characteristics

The nine included studies comprised 637 adult participants with BN, BED and EDNOS according to DSM criteria, of whom 283 were waitlisted. The interventions participants were waiting to receive encompassed various forms of psychotherapy delivered in weekly individual or group settings, including one GSH intervention [128] and one internet-based programme with personalised structured writing assignments and feedback [129]. Most of the studies were RCTs, except for one NRSI using a gender- and diagnosis-matched design [104]. All of the studies included participants with BED. EDNOS was represented in three studies [104, 128, 130] and BN in two studies [104, 130]. None of the studies included participants with AN.

Studies including participants with BN tended to report lower mean ages than studies without BN samples. The proportion of female participants ranged from 80.0% to 100.0%. Waiting periods varied between 4 and 16 weeks (9.89 weeks mean waiting period, 10.36 weeks sample-size weighted). Two studies did not report the full set of EDE-Q outcomes [102, 122]. One study only reported objective binge episodes (OBEs) and objective binge episode days (OBEDs) [131], as assessed using the six additional EDE-Q items not included in the Global Score. The missing information for these three studies could not be obtained by contacting the corresponding authors. None of the studies reported our secondary outcomes. Study characteristics are summarised in Table 1.

**Table 1:** Characteristics of included studies

First author, year	Country	Study design	Diagnostic manual	Diagnosis (%)	Conditions	Sample size WLCG (total)	Age group (range in years)	Mean age in years (SD)	Females in %	Duration of waiting in weeks	EDE-Q version	EDE-Q outcomes
Arcelus et al., 2012	United Kingdom	NRSI	DSM-IV	EDNOS (25.00) BN (58.30) BED (16.60)	IPT-BN10 IPT-BN16 WLCG	10 (30)	Adults (NR)	28.83 (7.80)	100.00	10	EDE-Q	GS, RE, WC, SC, EC
Berking et al., 2022	Germany	RCT	DSM-IV	BED (100.00)	ERST WLCG	40 (101)	Adults (18–69)	43.05 (12.81)	90.00	8	EDE-Q	GS, RE, WC, SC, EC
Fairburn et al., 2009	United Kingdom	RCT	DSM-IV	BN (38.30) EDNOS (61.70) BED (4.70 of the full sample)^a^	CBT-Ef CBT-Eb WLCG	51 (154)	Adults (18–65)	25.90 (6.40)	94.10	8	EDE-Q 6.0	GS, RE, WC, SC, EC
Glisenti et al., 2021	Australia	RCT	DSM-5	BED (100.00)	EFT WLCG	10 (21)	Adults (18–65)	45.80 (10.73)	80.00	12	EDE-Q	OBEs, OBEDs
Krohmer et al., 2022	Germany	RCT	DSM-5	BED (100.00)	ME WLCG	35 (72)	Adults (18–69)	44.34 (16.30)	100.00	4	EDE-Q	GS, WC, SC
Lewer et al., 2017	Germany	RCT	DSM-IV	BED (100.00)	CBT-BI WLCG	19 (34)	Adults (18–60)	NR	100.00	10	EDE-Q	RE, WC, SC, EC
Masson et al., 2013	Canada	RCT	DSM-IV	BED (NR) EDNOS (sub-BED) (NR)	DBT-GSH WLCG	30 (60)	Adults (NR)	43.43 (9.59)	86.70	13	EDE-Q 6.0	GS, RE, WC, SC, EC
Schlup et al., 2009	Switzerland	RCT	DSM-IV-TR	BED (100.00)	CBT-S WLCG	18 (36)	Adults (18–70)	41.20 (11.10)	100.00	8	EDE-Q	GS, RE, WC, SC, EC
Wagner et al., 2016	Germany	RCT	DSM-IV	BED (100.00)	iCBT WLCG	70 (139)	Adults (18–65)	35.30 (9.70)	98.60	16	EDE-Q	GS, RE, WC, SC, EC

All numbers refer to the WLCG only, unless stated otherwise

^a^Distribution of eating disorder diagnoses in the full sample, including the intervention group and the WLCG. Data were not reported separately for the WLCG

BED: binge-eating disorder; BN: bulimia nervosa; CBT-BI: Cognitive-Behavioural Body Image Therapy (group setting); CBT-Eb: enhanced Cognitive-Behavioural Therapy (broad form, individual setting); CBT-Ef : enhanced Cognitive-Behavioural Therapy (focused form, individual setting); CBT-S: shortened Cognitive-Behavioural Therapy followed by booster sessions after active treatment (group setting); DBT-GSH: guided self-help adaptation of Dialectical Behaviour Therapy (individual setting); DSM: Diagnostic and Statistical Manual of Mental Disorders; EC: Eating Concern; EDE-Q: Eating Disorder Examination-Questionnaire; EDNOS: eating disorder not otherwise specified; EFT: Emotion-Focused Therapy (individual setting); ERST: Emotion Regulation Skills Training (group setting); GS: Global Score; iCBT: internet-based therapist-supported Cognitive-Behavioural Therapy (individual setting); IPT-BN10: shortened Interpersonal Psychotherapy for Bulimia Nervosa (10 sessions, individual setting); IPT-BN16: conventional Interpersonal Psychotherapy for Bulimia Nervosa (16–20 sessions, individual setting); ME: guided stand-alone mirror exposure (individual setting); NR: not reported; NRSI: non-randomised study of intervention; OBEDs: objective binge episode days; OBEs: objective binge episodes; RE: Restraint; SC: Shape Concern; sub-BED: binge-eating disorder of low frequency and/or limited duration; WC: Weight Concern; WLCG: waiting list control group

Most studies excluded individuals with suicidal ideation (k = 7) [101, 102, 104, 122, 129, 131, 132], psychotic symptoms (k = 6) [101, 103, 128, 129, 131, 132] and SUDs (k = 5) [101, 102, 129, 131, 132]. Other psychiatric or medical reasons for exclusion included high levels of psychiatric comorbidity (k = 4) [101, 104, 129, 130], manic or bipolar symptoms (k = 3) [101, 102, 132], medical conditions affecting weight (k = 3) [102, 129, 132], borderline personality disorder (k = 2) [102, 132] or personality disorders in general (k = 1) [122], medical instability (k = 1) [130], deliberate self-harm (k = 1) [122], and intellectual disability (k = 1) [131]. Alongside concurrent psychotherapy (k = 7) [101, 102, 122, 128–130, 132], other relevant exclusion criteria were pregnancy (k = 7) [101, 102, 122, 129–132], reception of weight loss interventions (k = 4) [101, 129, 131, 132], (unstable) pharmacotherapeutic treatment (k = 3) [122, 128, 130], male sex (k = 2) [101, 122], and weight-affecting medication (k = 1) [132]. Among the studies that reported psychiatric comorbidities [101, 122, 130, 132], depressive and anxiety disorders were the most common diagnoses. Exclusion criteria, psychiatric comorbidities and BMI of the WLCGs are displayed in Additional file 8.

Where reported, participants were predominantly White/Caucasian/European, employed, received higher education and had a medium to high income. The place of residence, race, occupation, gender, religion, education, socioeconomic status, and social capital (PROGRESS) equity characteristics [133] for each included study are displayed in Table 1 and Additional file 9. Referring to the 'plus' characteristics [134, 135], *personal characteristics associated with discrimination*, i.e. age, psychiatric comorbidities and BMI, are included in Table 1 and Additional file 8. *Features of relationships* were not applicable. *Time-dependent relationships*, defined as instances where a person may be temporarily at a disadvantage, apply to all WLCG participants as they were waitlisted despite being in need of treatment.

The waitlist condition was defined similarly across RCTs, with participants randomised to the WLCG completing baseline assessments and then waiting for a set period until they were offered treatment. Measurements were repeated post-waiting, which was usually the same time point the IG finished treatment. One study repeated PRO measurement weekly [132], and one study described clinical monitoring during the waiting period [131]. Furthermore, two studies administered additional baseline measures, i.e. eye-tracking tasks and a digital photo distortion technique, alongside the semi-structured diagnostic interview and PROMs [102, 122]. In the NRSI by Arcelus et al. [104], data of individuals who waited to receive regular treatment at the treatment centre were used as controls. WLCGs descriptions are provided in Table 2.

**Table 2:** WLCG characteristics for each included study

First author, year	WLCG characteristics
Arcelus et al., 2012	Individuals who waited for ten weeks to receive regular treatment (i.e. IPT-BN16) at the Leicester Eating Disorders Service. No further procedures or contacts between baseline assessments (i.e. semi-structured interview and PROMs) and post-waiting assessments described
Berking et al., 2022	Participants randomised to the WLCG did not receive the intervention during the study but were offered treatment (i.e. ART or individual CBT) following the completion of all assessments after 8 weeks. Between baseline assessments (i.e. semi-structured interview and PROMs) and post-waiting assessments, weekly PROM measurements were administered
Fairburn et al., 2009	Participants randomised to the WLCG started treatment (i.e. CBT-Ef or CBT-Eb) with an 8-week delay. No further procedures or contacts between baseline assessments (i.e. semi-structured interview and PROMs) and post-waiting assessments are described
Glisenti et al., 2021	Participants randomised to the WLCG received treatment (i.e. EFT) after being waitlisted for 12 weeks. Between baseline assessments (i.e. semi-structured interview and PROMs) and post-waiting assessments, a 12-week clinical monitoring was carried out. This monitoring was not further specified
Krohmer et al., 2022	Participants randomised to the WLCG were offered treatment (i.e. ME) at the end of the study. No further procedures or contacts between baseline assessments (i.e. semi-structured interview, PROMs, and two experimental eye-tracking tasks) and post-waiting assessments are described. During the eye-tracking tasks, attention to the self-body relative to another body or an inanimate control stimulus, and to the most/least attractive body part was assessed
Lewer et al., 2017	Participants randomised to the WLCG waited for 10 weeks to receive treatment (i.e. CBT-BI). No further procedures or contacts between baseline assessments (i.e. semi-structured interview, PROMs, and a digital photo distortion technique) and post-waiting assessments are described. The digital photo distortion technique was applied to assess the perceptual component of body image disturbance by asking participants to adjust pictures of their own body to reflect perceived actual, felt, and ideal body size
Masson et al., 2013	Participants randomised to the WLCG received the treatment protocol (i.e. DBT-GSH) after 13 weeks. No further procedures or contacts between baseline assessments (i.e. semi-structured interview and PROMs) and post-waiting assessments are described
Schlup et al., 2009	Participants randomised to the WLCG entered the treatment condition (i.e. CBT-S) after completion of an 8-week waiting period. No further procedures or contacts between baseline assessments (i.e. semi-structured interview and PROMs) and post-waiting assessments are described
Wagner et al., 2016	Participants randomised to the WLCG started treatment (i.e. iCBT) after a 16-week waiting period. No further procedures or contacts between baseline assessments (i.e. semi-structured interview and PROMs) and post-waiting assessments are described

CBT-BI: Cognitive-Behavioural Body Image Therapy (group setting); CBT-Eb: enhanced Cognitive-Behavioural Therapy (broad form, individual setting); CBT-Ef: enhanced Cognitive-Behavioural Therapy (focused form, individual setting); CBT-S: shortened Cognitive-Behavioural Therapy followed by booster sessions after active treatment (group setting); DBT-GSH: guided self-help adaptation of Dialectical Behaviour Therapy (individual setting); EFT: Emotion-Focused Therapy (individual setting); ERST: Emotion Regulation Skills Training (group setting); iCBT: internet-based therapist-supported Cognitive-Behavioural Therapy (individual setting); IPT-BN10: shortened Interpersonal Psychotherapy for Bulimia Nervosa (10 sessions, individual setting); IPT-BN16: conventional Interpersonal Psychotherapy for Bulimia Nervosa (16–20 sessions, individual setting); ME: guided stand-alone mirror exposure (individual setting); PROM: patient-reported outcome measure; WLCG: waiting list control group

Potential conflicts of interest and funding sources of each included study were reviewed with no notable concerns regarding impact on study design and risk of bias in trial or synthesis result. Studies that were excluded due to ineligible PROMs particularly used the Binge Eating Scale [136] (k = 12), Eating Attitudes Test-26/-40 [137, 138] (k = 7), Eating Disorder Inventory [139] (k = 7), Bulimic Investigatory Test, Edinburgh [140] (k = 4) and Three-Factor Eating Questionnaire [141] (k = 4).

### Results of risk of bias assessment

**Fig. 3:**
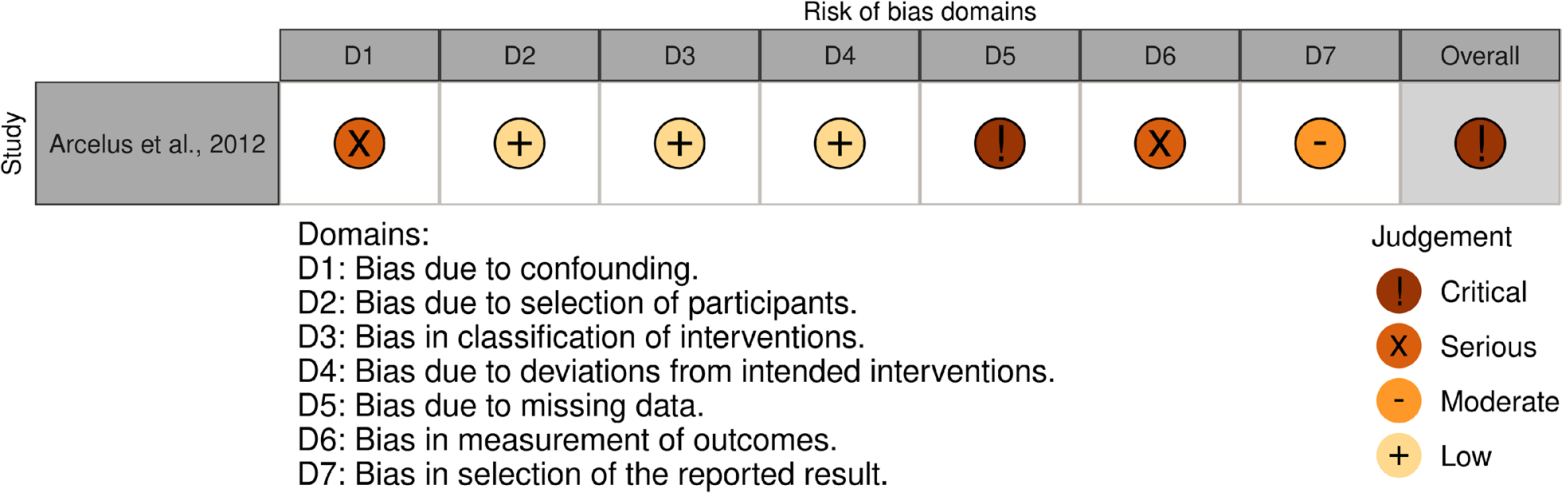
Risk of bias in the non-randomised study assessed with ROBINS-I. Traffic light plot generated using robvis [142]

Overall risk of bias was rated as high for all included RCTs (Fig. 2) and as critical for the one NRSI (Fig. 3). It is to be noted that in both RoB 2 (Domain 4) and ROBINS-I (Domain 6), bias in measurement of the outcome is, by definition, considered high when using PROMs in a wait-list controlled trial, as outcomes are self-assessed by participants who are aware of their group allocation. This knowledge is likely to influence outcome assessment, particularly given that participants in the IG are expected to show greater symptom improvement than those in the WLCG. Consequently, the overall risk of bias was rated as high by the algorithm, which defines the overall risk as high if at least one domain is judged to be at high risk.

Importantly, for Arcelus et al. [104], the critical risk judgement in Domain 5 resulted from uncertainty due to insufficient reporting. Had the signalling questions been answered solely based on information inferred from the reported tables, a low risk of bias would have been assigned, resulting in a serious rather than critical overall risk judgement. A conservative approach was chosen to ensure that uncertainty arising from incomplete reporting was appropriately reflected in the assessment. Detailed justifications for each assessment are provided in Additional file 10, Tables 1 and 2.

### Results of individual studies

Baseline EDE-Q mean scores varied considerably across studies, with Global Scores ranging from 3.08 to 4.65, Restraint from 1.82 to 4.14, Weight Concern from 3.52 to 5.12, Shape Concern from 4.06 to 5.45, and Eating Concern from 2.27 to 4.67. Across most studies, small decreases in symptoms were observed for the Global Score, Restraint, Weight Concern, and Eating Concern (maximum mean reductions of − 0.32, − 0.54, − 0.10, and – 0.93, respectively). In contrast, findings for Shape Concern were more ambiguous, with half of the studies reporting slight decreases (up to − 0.39) [102, 129, 130, 132] and the remainder reporting slight increases (up to 0.37) [101, 104, 122, 128]. Notable deviations included small increases across all EDE-Q scales except Eating Concern in Arcelus et al. [104], with mean changes ranging from 0.31 to 0.58, and minimal changes across all scales in Lewer et al. [122], with MDs ranging from – 0.06 to 0.05.

Glisenti et al. [131] was the only study to report EDE-Q OBEs and OBEDs over a 7-day reference period instead of the Global Score and subscales. Baseline OBEs were 5.10 (SD = 3.03), with no mean change observed post-waiting (MD = 0.00, SD = 2.94). Baseline OBEDs were 4.40 (SD = 2.41) and increased slightly at post-assessment (MD = 0.30, SD = 2.36).

EDE-Q Global Scores and subscales for the WLCGs are displayed in Additional file 11 Table 1, and EDE-Q OBEs and OBEDs for Glisenti et al. [131] in Additional file 11, Table 2. For contextualisation, it is noted that IGs were reported to show statistically significant pre–post reductions in ED symptoms or significantly greater improvements than waiting-list controls, whereas changes in the WLCG were little to absent [101, 102, 104, 122, 128, 130–132].

### Results of statistical analyses

**Fig. 4:**
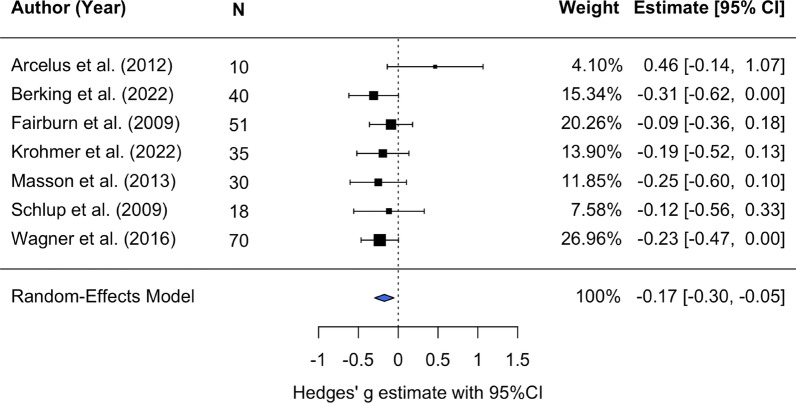
Forest plot for the primary analysis of the EDE-Q Global Score

Eight of the nine included studies (n = 273) reported the EDE-Q Global Score and subscales and were considered in the meta-analysis [101, 102, 104, 122, 128–130, 132]. Seven studies reported the Global Score and were included in the primary analysis [101, 102, 104, 128–130, 132]. The pooled effect estimate was Hedges’ g = – 0.17 (95% CI – 0.30 to – 0.05), indicating a small symptom reduction (Fig. 4).

**Fig. 5:**
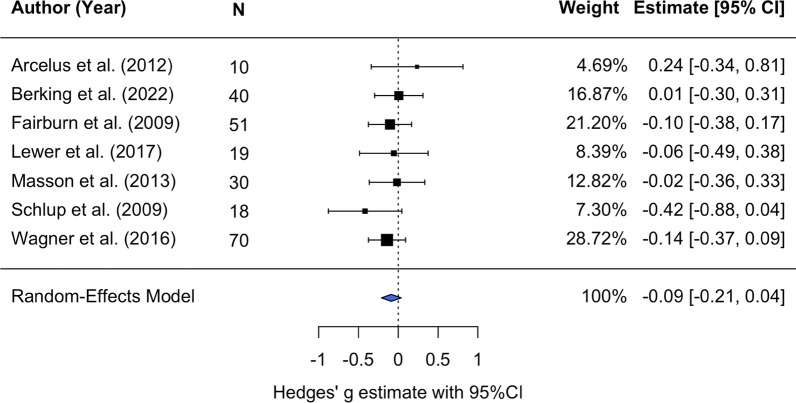
Forest plot for the secondary analysis of the EDE-Q subscale Restraint

**Fig. 6:**
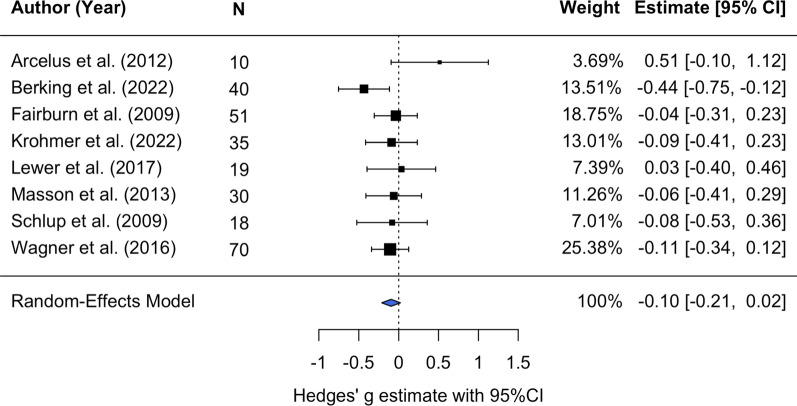
Forest plot for the secondary analysis of the EDE-Q subscale Weight Concern

**Fig. 7:**
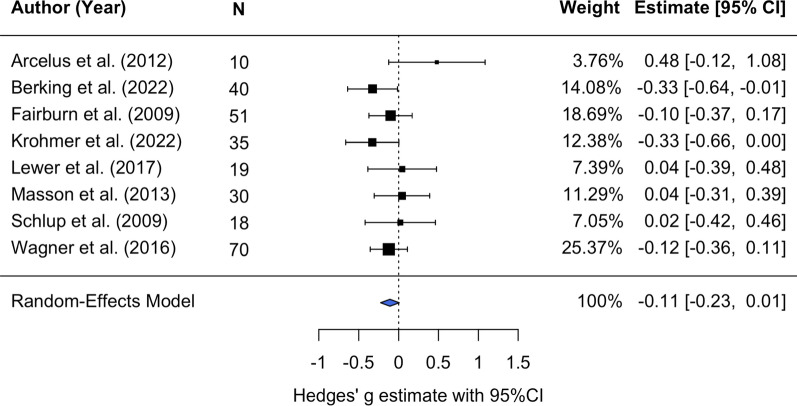
Forest plot for the secondary analysis of the EDE-Q subscale Shape Concern

**Fig. 8:**
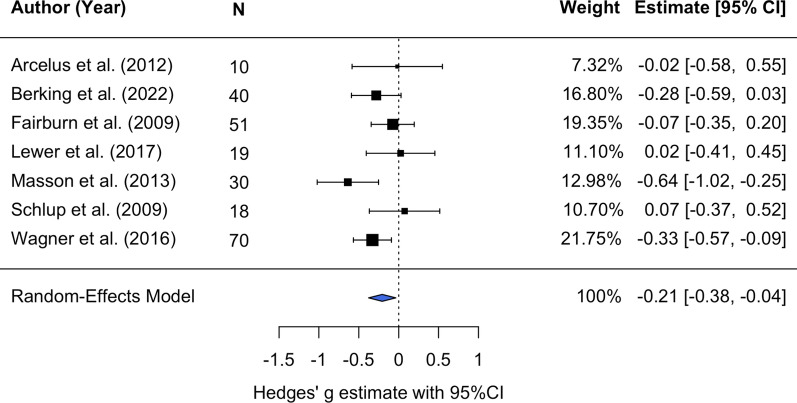
Forest plot for the secondary analysis of the EDE-Q subscale Eating Concern

Across the seven studies assessing Restraint [101, 104, 122, 128–130, 132], a small symptom reduction was found (Hedges’ g = − 0.09, 95% CI − 0.21 to 0.04) (Fig. 5). Weight Concern (Fig. 6) and Shape Concern (Fig. 7) scores were reported by eight studies [101, 102, 104, 122, 128–130, 132]. The pooled effect estimates were again small (Hedges’ g = − 0.10, 95% CI − 0.21 to 0.02, and Hedges’ g = − 0.11, 95% CI − 0.23 to 0.01, respectively). The largest pooled effect estimate was observed for Eating Concern [101, 104, 122, 128–130, 132]. Pooling the results of seven studies yielded a pooled effect estimate of Hedges’ g = − 0.21 (95% CI − 0.38 to − 0.04) (Fig. 8).

For the primary and most secondary analyses, heterogeneity was not detected or negligible (I^2^ = 0.00% for Restraint; I^2^ = 0.01% for the Global Score and Weight Concern; I^2^ = 0.02% for Shape Concern). In contrast, heterogeneity for Eating Concern was higher but still considered low (I^2^ = 39.53%). However, in small meta-analyses, I^2^ may underestimate heterogeneity by up to 28% [143].

#### Results of subgroup analyses

Subgroup analyses for waiting time revealed no large differences in Global Scores between waiting times of ≤ 10 weeks and > 10 weeks, with both showing small pooled reductions of the Global Score (Hedges' g = − 0.18, 95% CI − 0.34 to − 0.02, and Hedges' g = − 0.09, 95% CI − 0.44 to 0.26, respectively). Heterogeneity was not detected for shorter waiting times (I^2^ = 0.00%) but was substantial for longer waiting times (I^2^ = 62.49%).

Results varied slightly more in the subgroup analyses by diagnostic composition. The pooled Global Score symptom reduction in samples only including BED was Hedges' g = − 0.23 (95% CI − 0.38 to − 0.07), while it was smaller in mixed samples including BN, BED and EDNOS (Hedges' g = − 0.05, 95% CI − 0.35 to 0.25). Heterogeneity was not detected in studies with BED samples (I^2^ = 0.00%) and moderate in studies with mixed samples (I^2^ = 46.24%). Forest plots for the subgroup analyses are displayed in Additional file 12.

#### Results of sensitivity analyses

The sensitivity analysis excluding Arcelus et al. [104] resulted in a similar pooled effect estimate for the Global Score (Hedges' g = − 0.20, 95% CI − 0.33 to − 0.08). Excluding Krohmer et al. [102] in a further sensitivity analysis resulted in no change of the pooled Global Score symptom reduction (Hedges' g = − 0.17, 95% CI − 0.30 to − 0.04). In both analyses, heterogeneity was not detected (I^2^ = 0.00%).

Applying r = 0.8 as pre–post correlation coefficient resulted in a similar pooled Global Score symptom reduction (Hedges' g = − 0.21, 95% CI − 0.44 to 0.03), with substantial heterogeneity (I^2^ = 86.52%). Pooled Global Score symptom reductions were even smaller when applying r = 0.2 (Hedges' g = − 0.14, 95% CI − 0.29 to 0.02), with no detected heterogeneity (I^2^ = 0.00%). Forest plots for the sensitivity analyses are displayed in Additional file 13.

### Risk of publication bias

**Fig. 9:**
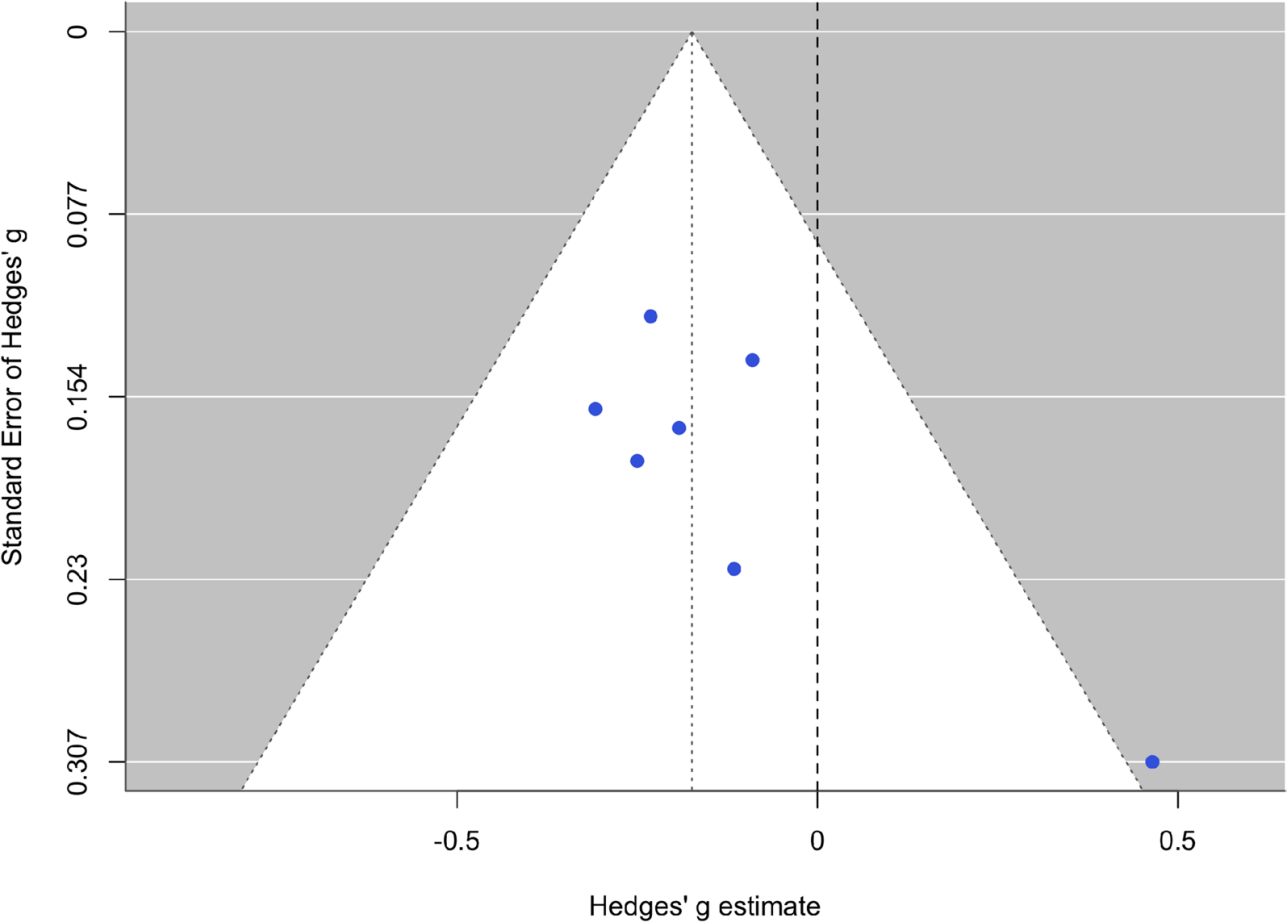
Funnel plot for the primary analysis of the EDE-Q Global Score

Visual inspection of the funnel plot for the primary analysis of the Global Score (Fig. 9) and the secondary analyses of the subscales did not suggest pronounced asymmetry. Most studies were distributed around the pooled effect estimate and lay within the pseudo-95% confidence limits. The minor asymmetry observed may be attributable to other factors, such as true heterogeneity or chance, and cannot reliably be interpreted as evidence for publication bias. For the secondary analyses of the subscales and the sensitivity analyses, the funnel plots can be found in Additional file 14.

### Certainty of evidence and critical appraisal

The certainty of evidence was rated as moderate for the EDE-Q outcomes Global Score, Eating Concern, OBEs and OBEDs, indicating that the true effect is probably close to the estimated effect. For the EDE-Q outcomes Restraint, Weight Concern and Shape Concern, the certainty of evidence was rated as low, indicating that the true effect may deviate from the estimated effect [144]. The downgrade was due to the high risk of bias in all included studies and possible imprecision, as indicated by the CIs for some of the outcomes. The 'Summary of Findings' for each outcome is presented in Table 3.

**Table 3:** Summary of Findings for each EDE-Q outcome

k	Study design	Certainty Assessment					Effect		Certainty
		Risk of bias	Inconsistency	Indirectness	Imprecision	Other considerations	n	Hedges' g (95% CI)	
Global Score, 28-day reference period (follow-up: range 4 to 16 weeks; assessed with: EDE-Q; scale from: 0 to 6)									
7	6 RCTs, 1 NRSI	Serious^a^	Not serious	Not serious	Not serious	None	257	− 0.17 (− 0.30 to − 0.05)	⊕ ⊕ ⊕ ◯ Moderate^a^
Restraint, 28-day reference period (follow-up: range 8 to 16 weeks; assessed with: EDE-Q; scale from: 0 to 6)									
7	6 RCTs, 1 NRSI	Serious^a^	Not serious	Not serious	Serious^b^	None	238	− 0.09 (− 0.21 to 0.04)	⊕ ⊕ ◯◯ Low^a,b^
Weight Concern, 28-day reference day period (follow-up: range 4 to 16 weeks; assessed with: EDE-Q; scale from: 0 to 6)									
8	7 RCTs, 1 NRSI	Serious^a^	Not serious	Not serious	Serious^b^	None	273	− 0.10 (− 0.21 to 0.02)	⊕ ⊕ ◯◯ Low^a,b^
Shape Concern, 28-day reference day period (follow-up: range 4 to 16 weeks; assessed with: EDE-Q; scale from: 0 to 6)									
8	7 RCTs, 1 NRSI	Serious^a^	Not serious	Not serious	Serious^b^	None	273	− 0.11 (− 0.23 to 0.01)	⊕ ⊕ ◯◯ Low^a,b^
Eating Concern, 28-day reference day period (follow-up: range 8 to 16 weeks; assessed with: EDE-Q; scale from: 0 to 6)									
7	6 RCTs, 1 NRSI	Serious^a^	Not serious	Not serious	Not serious	None	238	− 0.21 (− 0.38 to − 0.04)	⊕ ⊕ ⊕ ◯ Moderate^a^
Objective Binge Episodes, 7-day reference period (follow-up: 12 weeks; assessed with: EDE-Q)									
1	RCT	Serious^a^	Not serious	Not serious	Serious^c^	None	10	MD 0.00 (SD 2.94)	⊕ ⊕ ◯◯ Low^a,c^
Objective Binge Episode Days, 7-day reference period (follow-up: 12 weeks; assessed with: EDE-Q)									
1	RCT	Serious^a^	Not serious	Not serious	Serious^c^	None	10	MD 0.30 (SD 2.36)	⊕ ⊕ ◯◯ Low^a,c^

Table generated using GRADEpro GDT [145] and manually adapted

EDE-Q: Eating Disorder Examination-Questionnaire; k: number of studies; n: number of participants; NRSI: non-randomised study of intervention; RCT: randomised controlled trial

^a^The risk of bias was rated as high for outcome measurement across studies, which is inherent to PROMs and does not automatically imply poor study design. Other domains with risk of bias rated as high in some studies were deviations from intended intervention and missing outcome data. However, the risk was not deemed high enough for rating evidence down by two levels

^b^Confidence intervals include the possibility of no effect and aggravation of symptoms. Following a conservative approach, evidence was rated down by another level

^c^The confidence intervals were not calculated for OBEs and OBEDs as these outcomes were only reported by one study [131]. The downgrading of evidence was based on the small number of participants

The quality of our review was rated as high using the AMSTAR 2 tool, indicating an accurate and comprehensive summary of the results of the studies available on the review's objective [126]. Decisions for each item are provided in Additional file 15.

## Discussion

We systematically synthesised the available evidence on the effects of waiting time for outpatient psychotherapeutic interventions on PROs in adolescents and adults with EDs, using WLCGs. Nine studies including adults with BN, BED, and EDNOS met our eligibility criteria. Over a sample-size weighted mean waiting period of 10.36 weeks, ED pathology as assessed with the EDE-Q remained largely stable, with only small overall symptom reductions. Results from subgroup and sensitivity analyses were generally consistent with the main findings. Certainty of evidence was rated as low to moderate, predominantly given the high risk of bias across studies.

### Interpretation of results

Our findings align with previous meta-analyses in the context of social anxiety disorder and panic disorder [68, 69]. Importantly, the absence of aggravation does not equal an absence of burden. Baseline EDE-Q Global Scores across studies were considerably above established clinical cut-offs of 1.6 to 2.8, depending on the population [146–148], and comparable to other clinical samples [82, 146, 149–152].

While no symptom aggravation was observed, there was also no evidence of considerable improvement. Given the relatively short waiting periods, these findings should be interpreted in light of the predominantly chronic course of EDs. As participants entered the waiting period with high levels of ED pathology, symptoms may have stagnated at a clinically severe level. Thus, participants remained exposed to substantial distress, despite being treatment-seeking and eligible for intervention. A reduction in symptoms would have been expected if treatment had been initiated, either through allocation to the IG or timely access to routine care.

Importantly, our review refers to a treatment-seeking population, a group found to represent only 23.2% of the total ED population [153]. Seeking help requires insight into one's illness, as well as willingness to change and treatment motivation [62, 63, 154], which may distinguish participants from other affected individuals who might encounter different ED trajectories. Furthermore, individuals who deteriorated rapidly or required urgent care are most likely not participating in a WLCG.

There are a few general methodological considerations regarding the utilisation of WLCGs to take into account. Their use in psychological research has been criticised before, not only due to the possibility of a nocebo effect [155–157] but also because of ethical concerns when there already is an effective treatment. Nevertheless, WLCGs remain a standard control in psychological research [158]. However, experiencing waiting times in routine care and being allocated to a WLCG are not equivalent. In routine care, individuals seeking or waiting for outpatient psychotherapy do not know if or when they will get access. In contrast, participants in waitlist-controlled trials know exactly when and what form of treatment they will receive. This raises the question of the impact that knowledge of the exact waiting time and expectation of the intervention has on symptoms. The prospect of receiving the intervention could engender positive expectations [159], potentially reducing symptoms. The more extensive baseline assessment compared to routine care and the experience of being enrolled in an intervention study could also have a positive effect due to human contact and perceived social support [160], as well as increased hope and remoralisation [161, 162]. On the other hand, the frustration of not being allocated to the IG could lead to negative expectations, resulting in a nocebo effect and thereby accelerating symptoms [155–157].

For ethical reasons, waitlist conditions are usually not conducted for more extensive durations [129], which is why our research question cannot be answered for longer waiting times as commonly encountered in routine care [163]. We did not find large-scale studies evaluating routine or survey data as Reichert and Jacobs [66] and van Dijk et al. [67] did in the context of psychosis and major depressive disorder. Only the one NRSI by Arcelus et al. [104] used retrospective data from individuals awaiting regular treatment outside of the study context. Notably, they found the largest, albeit still small, increase in symptoms across studies.

### Limitations and strengths

Several limitations should be considered when interpreting our findings. Firstly, the actual duration of waiting remains unclear. Within this review, waiting time was defined as the number of weeks from study enrolment (baseline) to intervention start, with study enrolment representing a confirmed need for treatment based on a formal diagnosis. However, evidence also indicates substantial delays prior to this clinical indication and start of treatment: Most individuals seek help one to two years after the onset of symptoms [164, 165], and the reported DUED ranges from 2.5 years in AN up to 6 years in BED [65]. For most WLCGs, however, information on symptom duration and previous treatment was not available. Only two studies reported ED mean duration or mean age of first binge, suggesting several years of illness before study enrolment [130, 131]. Accordingly, WLCG participants likely experienced symptom onset several years prior to study entry, as well as earlier waiting periods and potentially psychotherapeutic treatment. Individuals may have initially experienced an accelerated deterioration of ED pathology, followed by sustained high symptomology over time [166]. As a result, early symptom changes associated with waiting for psychotherapy may not be captured, potentially underestimating the burden of treatment delay.

Secondly, the included study populations are not representative of the underlying ED target population. Notably, all included studies were conducted in Western industrial countries, potentially limiting the validity of our results. Participants were more likely to be White/Caucasian/European, female, and well educated, while Black, Indigenous, and People of Colour (BIPoC), as well as individuals with lower education and socioeconomic status were underrepresented. Studies that particularly examined interventions in BIPoC [167, 168] did not meet our eligibility criteria. Although EDs are more prevalent in females, the gender ratio is found to be more balanced in BED than in other EDs [169–171]. However, this was not reflected in the included studies, where males were underrepresented. Furthermore, it is unclear if and to what extent non-binary individuals were represented, given that studies used binary gender reporting.

We did not find eligible studies including participants with AN, which aligns with the findings of other meta-analyses [48, 54], nor studies including adolescents. Alongside waitlist controlled trials for AN being scarce, we excluded interventions that include caregivers to enhance comparability of studies. For adolescents with EDs, however, family interventions are an important and commonly used treatment, especially in the context of AN [22]. The representation of psychiatric comorbidities was limited due to differing exclusion diagnoses of individual studies. Notably, three studies excluded personality disorders in general, or borderline personality disorder specifically. However, personality disorders are highly prevalent in EDs at 50–70% [172, 173] and are a predictor of poorer ED outcomes [9].

Thirdly, there was methodological diversity across studies. Two studies included tasks with an exposure component in their baseline assessment [102, 122], for which we conducted sensitivity analyses. Not all studies reported exclusion or comorbid diagnoses, introducing uncertainty regarding sample composition. We set narrow eligibility criteria for WLCGs in order to mimic a waiting scenario in routine care as closely as possible. For the screening process, however, we had to establish a threshold for how much contact is permissible, which depended heavily on the description. For instance, we accepted medical monitoring, as described by Glisenti et al. [131], as we viewed it as a safety measure solely for ethical reasons (e.g. adverse health outcomes, suicide prevention), but we did not accept regular encouragement to continue waiting and reminders about receiving treatment afterwards, as described by Clyne et al. [174]. Lewer et al. [122] technically excluded individuals who received current psychotherapy but indicated that many of the WLCG participants started psychotherapy while being waitlisted. Although not reported in the other studies, we cannot be certain whether that might also has happened within other WLCGs. Thus, the generalisability may be impaired by unmeasured factors.

Finally, outcome measurement was limited to a single PROM, i.e. the EDE-Q. As the EDE-Q uses a 28-day reference period and waiting periods in approximately half of the studies lasted only 4 to 8 weeks, the instrument may not have been sensitive enough to detect short-term symptom changes. Other PROMs from the ICHOM set were not used in the eligible studies, restricting outcome assessment to ED pathology. Given the more recent publication of the ICHOM recommendations and the existence of a range of ED measures, other PROMs are frequently administered and could also be used to answer our research question. Despite their advantages, PROMs depend on individuals’ item comprehension [175] and self-awareness [131], and may be subject to non-response bias [176]. These factors may limit precision and sensitivity to change.

Taken together, these limitations reflect both methodological constraints within the existing literature as well as necessary methodological decisions in this review, such as the applied PROM set and clearly defined waiting conditions. These considerations inevitably resulted in a relatively small number of eligible studies. However, rather than indicating an overly narrow review scope, this primarily underscores the scarcity of comparable evidence on waiting time effects in EDs, particularly from a patient-centred perspective. At the same time, the application of these stricter criteria enabled a more consistent synthesis and the conduct of a meta-analysis, thereby strengthening the interpretability of our findings. Against this background, the present review should be understood not only as a synthesis of the available evidence but also as an identification of a substantial gap in the literature.

In spite of the shortcomings outlined above, our systematic review has several strengths. We conducted a comprehensive search without language restrictions to minimise the risk of reporting bias. We did not apply any date restrictions either. However, amending the eligibility of the primary instruments resulted in an implicit date restriction, given that the chEAT and EDE-Q were first published in 1988 and 1994, respectively. Nevertheless, the review covers the last three decades of ED research. The inclusion of OSFED addresses an often underrepresented diagnosis with a largely similar psychopathology and substantial mortality [11, 177]. Rather than using clinician-reported or BMI-centred outcome measurement, we used PROs to focus on the patients' experience of ED pathology. Finally, the PROM set was aligned with the recommendations of the ICHOM to ensure an international perspective and involvement of persons with lived experience.

### Implications for further research

Further research is necessary to gain insights into the effects of waiting times on PROs in children and adolescents with EDs. In this context, examining treatments involving caregivers as part of the support system is important to understand possible differences. Moreover, sub-threshold diagnoses as well as presentations of disordered eating that are not recognised in the DSM or ICD yet, such as orthorexia nervosa, should be considered, as they also come with a substantial burden [178, 179].

Future studies should also investigate the effectiveness of psychotherapy with delayed treatment initiation. While symptoms may not have worsened during the waiting period, waiting itself potentially contributes to chronicity, making later treatment less effective. This could lead to a prolongation of ED duration, which may also increase the risk of adverse outcomes and comorbidities [65, 180]. To rule out expectancy and nocebo effects, potentially leading to over- or underestimation of waiting time effects, it is essential to collect and evaluate routine data.

## Conclusion

This review provides evidence that short-term waiting times in adult WLCG participants with BN, BED and EDNOS are associated with persistently high ED pathology, as assessed with the EDE-Q. Although waiting time may not exacerbate symptoms in the short term, it nonetheless constitutes a clinically relevant delay in access to treatment. From a clinical perspective, waiting therefore represents a period during which substantial psychopathology and associated distress may persist without therapeutic intervention. Importantly, our results should be viewed in the context of WLCGs, which are not equivalent to waiting for treatment in routine care. The collection and analysis of routine data is essential to better understand the effects of waiting time for individuals awaiting regular treatment outside a study setting.

## Supplementary Information

Additional file 1. PECO framework.

Additional file 2. Completed PRISMA 2020 checklist.

Additional file 3. Rationales for exclusion diagnoses.

Additional file 4. Final search strings for all engines.

Additional file 5. Items for data extraction.

Additional file 6. Specification plots of Hedges’ g.

Additional file 7. Explanation for study exclusions.

Additional file 8. Exclusion criteria, psychiatric comorbidities and BMI for the WLCGs of included studies.

Additional file 9. PROGRESS equity characteristics for the WLCGs of included studies.

Additional file 10. Tables of Risk of bias assessment.

Additional file 11. EDE-Q Global and subscale scores, objective binge episodes and days for the WLCGs.

Additional file 12. Forest plots for subgroup analyses.

Additional file 13. Forest plots for sensitivity analyses.

Additional file 14. Funnel plots for secondary and sensitivity analyses.

Additional file 15. Decisions for each AMSTAR 2 item.

## Data Availability

The datasets used and/or analysed during the current study are available from the corresponding author on reasonable request. The same applies to the R code.

## Abbreviations

AAN: Atypical anorexia nervosa
AMSTAR: A MeaSurement Tool to Assess systematic Reviews
AN: Anorexia nervosa
ARFID: Avoidant/restrictive food intake disorder
BASE: Bielefeld Academic Search Engine
BED: Binge-eating disorder
BIPoC: Black, Indigenous and People of Colour
BMI: Body mass index
BN: Bulima nervosa
CBT: Cognitive behavioural therapy
CENTRAL: Cochrane Central Register of Controlled Trials
chEAT: Children's Eating Attitudes Test
CI: Confidence interval
DSM: Diagnostic and Statistical Manual of Mental Disorders
DUED: Duration of untreated eating disorder
EDNOS: Eating disorder not otherwise specified
ED: Eating disorder
EDE: Eating disorder examination
EDE-Q: Eating disorder examination questionnaire
GSH: Guided self help
GRADE: Grading of Recommendations, Assessment, Development and Evaluation
ICD: International Statistical Classification of Diseases and Related Health Problems
ICHOM: International Consortium for Health Outcomes Measurement
IG: Intervention group
k: Number of studies
MD: Mean difference
n: Number of participants
NES: Night eating syndrome
NRSI: Non-randomised study of intervention
OBE: Objective binge episode
OBED: Objective binge episode day
OSFED: Other specified feeding or eating disorder
PECO: Population, exposure, comparator, outcome
PD: Purging disorder
PRISMA: Preferred Reporting Items for Systematic reviews and Meta-Analyses
PRO: Patient-reported outcome
PROGRESS: Place of residence, race, occupation, gender, religion, education, socioeconomic status, and social capital
PROM: Patient-reported outcome measure
QoL: Quality of life
RCT: Randomised controlled trial
RD: Rumination disorder
RoB 2: Cochrane Risk of Bias 2
ROBINS-I: Risk Of Bias In Non-randomised Studies of Interventions
SD: Standard deviation
SMR: Standardised mortality ratio
sub-BED: Binge-eating disorder of low frequency and/or limited duration
sub-BN: Bulimia nervosa of low frequency and/or limited duration
SUD: Substance use disorder
SWiM: Synthesis Without Meta-analysis
TERA: The Evidence Review Accelerator
UFED: Unspecified feeding or eating disorder
WHO: World Health Organization
WLCG: Waitlist control group
