# Supplementary material for: The effects of waiting time for outpatient psychotherapeutic interventions on patient-reported outcomes in adolescents and adults with eating disorders: a systematic review and meta-analysis
Source: J Eat Disord. 2026 Jun 5;14:129. doi: 10.1186/s40337-026-01660-4 (PMC13248287; doi:10.1186/s40337-026-01660-4)
Supplement: Supplementary file 1 — Additional file 1. PECO framework. [file 40337_2026_1660_MOESM1_ESM.pdf]

Additional file 1

Table | PECO framework.

Element	Definition
Population	Adolescents (according to the WHO[1] definition, i.e. 10-19 years) and adults (≥ 18 years) with AN, BN, BED or OSFED, according to DSM or ICD (research) criteria that were valid at the time of diagnosis, or who exceeded validated cut-off values in questionnaires or interviews based on DSM or ICD (research) criteria for AN, BN, BED or OSFED that are explicitly stated to be used as a proxy for full diagnosis
Exposure	Waiting time for an outpatient psychotherapeutic intervention, defined as the number of days or weeks from initial consultation, diagnostic workup, or study enrolment (baseline) to intervention start
Comparator	Optional, depending on the study design; for comparative studies, an active psychotherapeutic intervention in an outpatient setting
Outcomes	*Primary Outcomes:* ED-specific PROs, measured using the Children's Eating Attitudes Test[2], Eating Disorder Examination-Questionnaire[3] or Eating Disorder Examination-Questionnaire 6.0[4]
	Secondary Outcomes: Generic PROs of well-being or functioning (measured using the KIDSCREEN-10[5] or the WHO Disability Assessment Schedule 2.0[6]), other mental health-related PROs, i.e. measures of anxiety, depression, and suicidal ideation, (measured using the Revised Children's Anxiety and Depression Scale-25[7], Patient Health Questionnaire-2[8], Patient Health Questionnaire-9[9], Generalised Anxiety Disorder-2 or Generalised Anxiety Disorder-7[10]), as well as ED-specific QoL and social functioning, measured using the Clinical Impairment Assessment[11]

Note: AN = anorexia nervosa; BED = binge-eating disorder; BN = bulimia nervosa; DSM = Diagnostic and Statistical Manual of Mental Disorders; ED = eating disorder; ICD = International Statistical Classification of Diseases and Related Health Problems; OSFED = other specified feeding or eating disorder; PRO = patient-reported outcome; QoL = Quality of Life; WHO = World Health Organisation.

Reference List

1. World Health Organization. Adolescent health [Internet]. 2025. https://www.who.int/health-topics/adolescent-health/#tab=tab_1

2. Maloney MJ, McGUIRE JB, Daniels SR. Reliability Testing of a Children’s Version of the Eating Attitude Test. Journal of the American Academy of Child & Adolescent Psychiatry. 1988;27:541–3. https://doi.org/10.1097/00004583-198809000-00004

3. Fairburn CG, Beglin SJ. Eating Disorder Examination Questionnaire [Internet]. 1994 [cited 2025 Oct 6]. https://doi.org/10.1037/t03974-000

4. Fairburn CG, Beglin SJ. Eating Disorder Examination Questionnaire (EDE-Q 6.0). Cognitive behavior therapy and eating disorders. New York: Guilford Press; 2008. p. 309–13.

5. Ravens-Sieberer U, Erhart M, Rajmil L, Herdman M, Auquier P, Bruil J, et al. Reliability, construct and criterion validity of the KIDSCREEN-10 score: a short measure for children and adolescents’ well-being and health-related quality of life. Qual Life Res. 2010;19:1487–500. https://doi.org/10.1007/s11136-010-9706-5

6. Üstün TB, Kostanjsek N, Chatterji S, Rehm J. Measuring Health and Disability: Manual for WHO Disability Assessment Schedule (WHODAS 2.0). Geneva: World Health Organization; 2010.

7. Ebesutani C, Korathu-Larson P, Nakamura BJ, Higa-McMillan C, Chorpita B. The Revised Child Anxiety and Depression Scale 25–Parent Version: Scale Development and Validation in a School-Based and Clinical Sample. Assessment. 2017;24:712–28. https://doi.org/10.1177/1073191115627012

8. Kroenke K, Spitzer RL, Williams JBW. The Patient Health Questionnaire-2: Validity of a Two-Item Depression Screener. Medical Care. 2003;41:1284–92. https://doi.org/10.1097/01.MLR.0000093487.78664.3C

9. Kroenke K, Spitzer RL, Williams JBW. The PHQ-9: Validity of a brief depression severity measure. J Gen Intern Med. 2001;16:606–13. https://doi.org/10.1046/j.1525-1497.2001.016009606.x

10. Spitzer RL, Kroenke K, Williams JBW, Löwe B. A Brief Measure for Assessing Generalized Anxiety Disorder: The GAD-7. Arch Intern Med. 2006;166:1092. https://doi.org/10.1001/archinte.166.10.1092

11. Bohn K, Doll HA, Cooper Z, O'Connor M, Palmer RL, Fairburn CG. The measurement of impairment due to eating disorder psychopathology. Behaviour Research and Therapy. 2008;46:1105–10. https://doi.org/10.1016/j.brat.2008.06.012
