# Supplementary material for: The effects of waiting time for outpatient psychotherapeutic interventions on patient-reported outcomes in adolescents and adults with eating disorders: a systematic review and meta-analysis
Source: J Eat Disord. 2026 Jun 5;14:129. doi: 10.1186/s40337-026-01660-4 (PMC13248287; doi:10.1186/s40337-026-01660-4)
Supplement: Supplementary file 3 — Additional file 3. Rationales for exclusion diagnoses. [file 40337_2026_1660_MOESM3_ESM.pdf]

Additional file 3

Rationales for exclusion diagnoses

Feeding disorders, i.e. 'avoidant/restrictive food intake disorder', pica and rumination disorder, were excluded as they primarily onset and occur in childhood. While adolescents and adults can also be affected, these cases are often associated with neurodevelopmental disorders,[1] which may impair the ability to report symptoms accurately and thus reduce the validity of patient-reported outcomes. Moreover, an association between pica and schizophrenia has been described.[1–3] 'Unspecified feeding or eating disorder' was excluded as it is usually diagnosed in situations with insufficient information, such as in emergency room settings,[1] thus lacking diagnostic specificity.[4] Bipolar disorders were excluded due to the potential for manic episodes to distort measurement of eating disorder outcomes.[5] Psychotic disorders and active substance use disorders were excluded, as impaired reality testing or cognitive impairment could compromise the validity of patient-reported outcomes.[6,7] However, this does not apply to nicotine dependence,[8] hence it is not listed as an exclusion diagnosis.

To enhance the generalisability of our findings, studies including participants with other comorbidities were included. This approach reflects the high prevalence of psychiatric[9] and somatic comorbidities[10,11] in routine care and allows for findings that are more representative of clinical practice. For the same reason we allowed stable pharmacotherapeutic medication that was not part of the study intervention. Forms of therapy distinct from outpatient psychotherapeutic interventions, as defined in our eligibility criteria, were excluded to ensure comparability between the populations awaiting treatment across studies.

Reference List

1. American Psychiatric Association, editor. Diagnostic and statistical manual of mental disorders: DSM-5. 5th ed. Washington, D.C: American Psychiatric Association; 2013.

2. Kouidrat Y, Amad A, Lalau J-D, Loas G. Eating Disorders in Schizophrenia: Implications for Research and Management. Schizophrenia Research and Treatment. 2014;2014:1–7. https://doi.org/10.1155/2014/791573

3. Osuji PN, Onu JU. Feeding behaviors among incident cases of schizophrenia in a psychiatric hospital: Association with dimensions of psychopathology and social support. Clinical Nutrition ESPEN. 2019;34:125–9. https://doi.org/10.1016/j.clnesp.2019.08.001

4. Ekeroth K, Clinton D, Norring C, Birgegård A. Clinical characteristics and distinctiveness of DSM-5 eating disorder diagnoses: findings from a large naturalistic clinical database. J Eat Disord. 2013;1:31. https://doi.org/10.1186/2050-2974-1-31

5. Phillips ML, Kupfer DJ. Bipolar disorder diagnosis: challenges and future directions. The Lancet. 2013;381:1663–71. https://doi.org/10.1016/S0140-6736(13)60989-7

6. Lee JS, Chun JW, Lee S, Kang D-I, Kim J-J. Association of impaired reality processing with psychotic symptoms in schizophrenia. Psychiatry Research. 2013;210:721–8. https://doi.org/10.1016/j.psychres.2013.07.035

7. Ramey T, Regier PS. Cognitive impairment in substance use disorders. CNS Spectr. 2019;24:102–13. https://doi.org/10.1017/S1092852918001426

8. Valentine G, Sofuoglu M. Cognitive Effects of Nicotine: Recent Progress. CN. 2018;16:403–14. https://doi.org/10.2174/1570159X15666171103152136

9. Kessler RC, Chiu WT, Demler O, Walters EE. Prevalence, Severity, and Comorbidity of 12-Month DSM-IV Disorders in the National Comorbidity Survey Replication. Arch Gen Psychiatry. 2005;62:617. https://doi.org/10.1001/archpsyc.62.6.617

10. Meuret AE, Tunnell N, Roque A. Anxiety Disorders and Medical Comorbidity: Treatment Implications. In: Kim Y-K, editor. Anxiety Disorders [Internet]. Singapore: Springer Singapore; 2020 [cited 2025 June 9]. p. 237–61. https://doi.org/10.1007/978-981-32-9705-0_15

11. Read JR, Sharpe L, Modini M, Dear BF. Multimorbidity and depression: A systematic review and meta-analysis. Journal of Affective Disorders. 2017;221:36–46. https://doi.org/10.1016/j.jad.2017.06.009
