# Supplementary material for: The effects of waiting time for outpatient psychotherapeutic interventions on patient-reported outcomes in adolescents and adults with eating disorders: a systematic review and meta-analysis
Source: J Eat Disord. 2026 Jun 5;14:129. doi: 10.1186/s40337-026-01660-4 (PMC13248287; doi:10.1186/s40337-026-01660-4)
Supplement: Supplementary file 4 — Additional file 4. Final search strings for all engines. [file 40337_2026_1660_MOESM4_ESM.pdf]

Additional file 4

Final search strings for all engines executed on 12 August 2025

Ovid MEDLINE(R) ALL <1946 to August 11, 2025>

1	exp "Feeding and Eating Disorders"/	39461
2	eating disorder*.ab,kf,ti.	30182
3	anorexia nervosa.ab,kf,ti.	17505
4	bulimia nervosa.ab,kf,ti.	7041
5	binge eating disorder.ab,kf,ti.	3926
6	"other specified feeding or eating disorder*".ab,kf,ti.	113
7	OSFED.ab,kf,ti.	123
8	"eating disorder* not otherwise specified".ab,kf,ti.	639
9	EDNOS.ab,kf,ti.	397
10	purging disorder.ab,kf,ti.	129
11	night eating syndrome.ab,kf,ti.	365
12	1 or 2 or 3 or 4 or 5 or 6 or 7 or 8 or 9 or 10 or 11	53183
13	Waiting Lists/	14815
14	wait*.ab,kw,ti.	82833
15	exp Time-to-Treatment/	11609
16	delay*.ab,kw,ti.	640481
17	13 or 14 or 15 or 16	726700
18	Control Groups/	2152
19	control group.ab,kw,ti.	580769
20	comparison group.ab,kw,ti.	18997
21	comparator.ab,kw,ti.	16787
22	untreated group.ab,kw,ti.	6558
23	no-treatment group.ab,kw,ti.	1447
24	18 or 19 or 20 or 21 or 22 or 23	622531
25	17 or 24	1326784
26	exp Psychotherapy/	233429
27	psychotherap*.ab,kf,ti.	59551
28	counsel?ing.ab,kf,ti.	135213
29	psychoeducation.ab,kf,ti.	5747
30	exp Therapeutics/	5543755
31	therap*.ab,kw,ti.	3945314
32	treatment*.ab,kw,ti.	6190964
33	intervention*.ab,kw,ti.	1625616
34	26 or 27 or 28 or 29 or 30 or 31 or 32 or 33	12413059
35	12 and 25 and 34	1736

Embase Classic+Embase <1947 to 2025 August 08>

1	exp eating disorder/	78458
2	eating disorder*.ab,kf,ti.	40208
3	anorexia nervosa.ab,kf,ti.	23075
4	bulimia nervosa.ab,kf,ti.	9179
5	binge eating disorder.ab,kf,ti.	5083

6	"other specified feeding or eating disorder*".ab,kf,ti.	139
7	OSFED.ab,kf,ti.	144
8	"eating disorder* not otherwise specified".ab,kf,ti.	833
9	EDNOS.ab,kf,ti.	546
10	purging disorder.ab,kf,ti.	152
11	night eating syndrome.ab,kf,ti.	519
12	1 or 2 or 3 or 4 or 5 or 6 or 7 or 8 or 9 or 10 or 11	84002
13	wait*.ab,kw,ti.	142613
14	"time to treatment"/	32684
15	therapy delay/	22659
16	delay*.ab,kw,ti.	935412
17	13 or 14 or 15 or 16	1095326
18	control group/	122208
19	control group.ab,kw,ti.	890839
20	comparison group.ab,kw,ti.	26684
21	comparator.ab,kw,ti.	31771
22	untreated group.ab,kw,ti.	9556
23	no-treatment group.ab,kw,ti.	2141
24	18 or 19 or 20 or 21 or 22 or 23	972375
25	17 or 24	2029345
26	exp psychotherapy/	377536
27	psychotherap*.ab,kf,ti.	92428
28	psychological counseling/	1823
29	counsel?ing.ab,kf,ti.	208152
30	psychoeducation/	16174
31	psychoeducation.ab,kf,ti.	9794
32	exp therapy/	12824663
33	therap*.ab,kw,ti.	6070182
34	treatment*.ab,kw,ti.	9270066
35	early intervention/	42066
36	intervention*.ab,kw,ti.	2347334
37	26 or 27 or 28 or 29 or 30 or 31 or 32 or 33 or 34 or 35 or 36	19633440
38	12 and 25 and 37	3660

CENTRAL

ID	Search	Hits
#1	MeSH descriptor: [Feeding and Eating Disorders] explode all trees	2640
#2	("eating disorder"):ti,ab,kw	2967
#3	("anorexia nervosa"):ti,ab,kw	1482
#4	("bulimia nervosa"):ti,ab,kw	1000
#5	("binge eating disorder"):ti,ab,kw	1077
#6	("other specified feeding or eating disorder"):ti,ab,kw	8
#7	(OSFED):ti,ab,kw	18
#8	("eating disorder not otherwise specified"):ti,ab,kw	61
#9	(EDNOS):ti,ab,kw	48
#10	("purging disorder"):ti,ab,kw	16
#11	("night eating syndrome"):ti,ab,kw	24
#12	#1 OR #2 OR #3 OR #4 OR #5 OR #6 OR #7 OR #8 OR #9 OR #10 OR #11	4793

#13 MeSH descriptor: [Waiting Lists] this term only 828
#14 (wait):ti,ab,kw 8258
#15 MeSH descriptor: [Time-to-Treatment] explode all trees 721
#16 (delay):ti,ab,kw 18008
#17 #13 OR #14 OR #15 OR #1627167
#18 MeSH descriptor: [Control Groups] this term only 334
#19 ("control group"):ti,ab,kw 304726
#20 ("comparison group"):ti,ab,kw 4522
#21 (comparator):ti,ab,kw 12809
#22 ("untreated group"):ti,ab,kw 769
#23 ("no treatment group"):ti,ab,kw 704
#24 #18 OR #19 OR #20 OR #21 OR #22 OR #23 321669
#25 #17 OR #24 342767
#26 MeSH descriptor: [Psychotherapy] explode all trees 36928
#27 (psychotherapy):ti,ab,kw 17145
#28 (counseling):ti,ab,kw 29579
#29 (psychoeducation):ti,ab,kw 5396
#30 MeSH descriptor: [Therapeutics] explode all trees 432329
#31 (therapy):ti,ab,kw 923529
#32 (treatment):ti,ab,kw 1018064
#33 (intervention):ti,ab,kw 618800
#34 #26 OR #27 OR #28 OR #29 OR #30 OR #31 OR #32 OR #33 1673335
#35 #12 AND #25 AND #34 692

APA PsycInfo (via EBSCO)

((DE "Eating Disorders" OR DE "Anorexia Nervosa" OR DE "Avoidant/Restrictive Food Intake Disorder" OR DE "Binge Eating Disorder" OR DE "Bulimia" OR DE "Feeding Disorders" OR DE "Hyperphagia" OR DE "Kleine Levin Syndrome" OR DE "Orthorexia" OR DE "Pica" OR DE "Purging (Eating Disorders)" OR DE "Rumination (Eating)") OR XB (eating disorder* OR anorexia nervosa OR bulimia nervosa OR binge eating disorder OR "other specified feeding or eating disorder*" OR OSFED OR "eating disorder* not otherwise specified" OR EDNOS OR purging disorder OR night eating syndrome)) AND XB (wait* OR delay* OR control group OR comparison group OR comparator OR untreated group OR no-treatment group) AND ((DE "Psychotherapy" OR DE "Adlerian Psychotherapy" OR DE "Adolescent Psychotherapy" OR DE "Affirmative Therapy" OR DE "Analytical Psychotherapy" OR DE "Autogenic Training" OR DE "Brief Psychotherapy" OR DE "Brief Relational Therapy" OR DE "Child Psychotherapy" OR DE "Client Centered Therapy" OR DE "Compassion Focused Therapy" OR DE "Couples Therapy" OR DE "Drama Therapy" OR DE "Eclectic Psychotherapy" OR DE "Educational Therapy" OR DE "Emotion Focused Therapy" OR DE "Existential Therapy" OR DE "Experiential Psychotherapy" OR DE "Expressive Psychotherapy" OR DE "Eye Movement Desensitization Therapy" OR DE "Feminist Therapy" OR DE "Geriatric Psychotherapy" OR DE "Gestalt Therapy" OR DE "Group Psychotherapy" OR DE "Guided Imagery" OR DE "Humanistic Psychotherapy" OR DE "Hypnotherapy" OR DE "Individual Psychotherapy" OR DE "Insight Therapy" OR DE "Integrative Psychotherapy" OR DE "Interpersonal Psychotherapy" OR DE "Logotherapy" OR DE "Metacognitive Therapy" OR DE "Narrative Therapy" OR DE "Network Therapy" OR DE "Personal Therapy" OR DE "Persuasion Therapy" OR DE "Positive Psychology Therapy" OR DE "Primal Therapy" OR DE "Psychoanalysis" OR DE "Psychodrama" OR DE "Psychodynamic Psychotherapy" OR DE "Psychotherapeutic

Counseling" OR DE "Psychotherapeutic Techniques" OR DE "Rational Emotive Behavior Therapy" OR DE "Reality Therapy" OR DE "Relationship Therapy" OR DE "Solution Focused Therapy" OR DE "Spiritually Oriented Therapy" OR DE "Strategic Therapy" OR DE "Supportive Psychotherapy" OR DE "Transactional Analysis") OR DE "Psychoeducation" OR (DE "Treatment" OR DE "Addiction Treatment" OR DE "Adjunctive Treatment" OR DE "Aftercare" OR DE "Alternative Medicine" OR DE "Anxiety Management" OR DE "Behavior Therapy" OR DE "Bibliotherapy" OR DE "Brief Interventions" OR DE "Caregiving" OR DE "Client Transfer" OR DE "Client Treatment Matching" OR DE "Computer Assisted Therapy" OR DE "Conversion Therapy" OR DE "Counseling" OR DE "Creative Arts Therapy" OR DE "Cross Cultural Treatment" OR DE "Culturally Adapted Interventions" OR DE "Electronic Health Services" OR DE "Exercise Therapy" OR DE "Habilitation" OR DE "Health Care Services" OR DE "Human Potential Movement" OR DE "Human Services" OR DE "Institutionalization" OR DE "Integrated Services" OR DE "Interdisciplinary Treatment Approach" OR DE "Intervention" OR DE "Involuntary Treatment" OR DE "Life Sustaining Treatment" OR DE "Maintenance Therapy" OR DE "Medical Treatment (General)" OR DE "Mentalization-Based Interventions" OR DE "Milieu Therapy" OR DE "Mind Body Therapy" OR DE "Mindfulness-Based Interventions" OR DE "Movement Therapy" OR DE "Multimodal Treatment Approach" OR DE "Multisystemic Therapy" OR DE "Nature-Based Interventions" OR DE "Outpatient Treatment" OR DE "Pain Management" OR DE "Physical Treatment Methods" OR DE "Private Practice" OR DE "Psychoeducation" OR DE "Psychosocial Interventions" OR DE "Psychotherapy" OR DE "Recreation Therapy" OR DE "Rehabilitation" OR DE "Respite Care" OR DE "Self-Help Techniques" OR DE "Sex Therapy" OR DE "Social Services" OR DE "Sociotherapy" OR DE "Strengths-Based Interventions" OR DE "Stress Management" OR DE "Symptoms Based Treatment" OR DE "Therapeutic Processes" OR DE "Transdiagnostic Treatment" OR DE "Trauma-Informed Care" OR DE "Trauma Treatment" OR DE "Treatment Guidelines" OR DE "Treatment Outcomes" OR DE "Treatment Planning" OR DE "Video-Based Interventions" OR DE "Youth Services") OR XB (psychotherap* OR counsel#ing OR psychoeducation OR therap* OR treament* OR intervention))

BASE
subj:("eating disorders" OR "anorexia nervosa" OR "bulimia nervosa" OR "binge eating disorder" OR "other specified feeding or eating disorder" OR OSFED OR "eating disorder not otherwise specified" OR EDNOS OR "purging disorder" OR "night eating syndrome") AND (wait* OR delay* OR "control group" OR "comparison group" OR comparator OR "untreated group" OR "no treatment group") AND (psychotherap* OR counseling OR counselling OR psychoeducation OR therap* OR treatment* OR intervention*) doctype:(12* 13 14 15 18* 19 F)
