# Supplementary material for: The effects of waiting time for outpatient psychotherapeutic interventions on patient-reported outcomes in adolescents and adults with eating disorders: a systematic review and meta-analysis
Source: J Eat Disord. 2026 Jun 5;14:129. doi: 10.1186/s40337-026-01660-4 (PMC13248287; doi:10.1186/s40337-026-01660-4)
Supplement: Supplementary file 5 — Additional file 5. Items for data extraction. [file 40337_2026_1660_MOESM5_ESM.pdf]

Additional file 5

Table | Items for data extraction.

Category		Data Items	
1.	Population	1.1	(Mean) age
		1.2	Sex/Gender
		1.3	Ethnicity
		1.4	ED diagnosis
		1.5	Subpopulation (e.g. severe and enduring EDs)
		1.6	Diagnostic instrument(s) and diagnostic manual (DSM or ICD)
		1.7	Psychiatric comorbidities
		1.8	Other PROGRESS-Plus equity characteristics
2.	Exposure	2.1	Definition of waiting time
		2.2	Waiting time (in days/weeks)
3.	Comparator	3.1	Type of psychotherapeutic intervention
		3.2	Provider of psychotherapeutic intervention
		3.3	Duration of psychotherapeutic intervention (in sessions/weeks)
		3.4	Individual or group setting
		3.5	Mode of delivery (e.g. in-person, online, app-based)
4.	Outcomes	4.1	ED-specific PROs (incl. instruments)
		4.2	Generic PROs (incl. instruments)
		4.3	Other mental health-related PROs (incl. instruments)
		4.4	Time points of measurement
		4.5	Effect estimates/effect sizes
		4.6	P values
5.	Study Characteristics	5.1	Authors
		5.2	Year of publication
		5.3	Country
		5.4	Study design
		5.5	Aim of study
		5.6	Study setting/Care setting
		5.7	Sample size
		5.8	Randomisation

Note: DSM = Diagnostic and Statistical Manual of Mental Disorders; ED = eating disorder; ICD = International Statistical Classification of Diseases and Related Health Problems; PRO = patient-reported outcome; PROGRESS = place of residence, race, occupation, gender, religion, education, socioeconomic status, and social capital[1]. The 'plus' characteristics refer to *personal characteristics associated with discrimination*, *features of relationships* and *time-dependent relationships*.[2,3]

Reference List

1. O'Neill J, Tabish H, Welch V, Petticrew M, Pottie K, Clarke M, et al. Applying an equity lens to interventions: using PROGRESS ensures consideration of socially stratifying factors to illuminate inequities in health. Journal of Clinical Epidemiology. 2014;67:56–64. https://doi.org/10.1016/j.jclinepi.2013.08.005

2. Oliver S, Kavanagh J, Caird J, Lorenc T, Oliver K, Harden A, et al. Health promotion, inequalities and young people’s health: a systematic review of research ; report. London: EPPI Centre, Social Science Research Unit, Institute of Education, University of London; 2008.

3. Oliver S, Dickson K, Newmann M. Getting started with a review. In: Gough D, Oliver S, Thomas J, editors. An Introduction to Systematic Reviews. London: SAGE Publications; 2012.
