# Supplementary material for: The effects of waiting time for outpatient psychotherapeutic interventions on patient-reported outcomes in adolescents and adults with eating disorders: a systematic review and meta-analysis
Source: J Eat Disord. 2026 Jun 5;14:129. doi: 10.1186/s40337-026-01660-4 (PMC13248287; doi:10.1186/s40337-026-01660-4)
Supplement: Supplementary file 6 — Additional file 6. Specification plots of Hedges’ g. [file 40337_2026_1660_MOESM6_ESM.pdf]

Additional file 6

Specification plots of Hedges' g

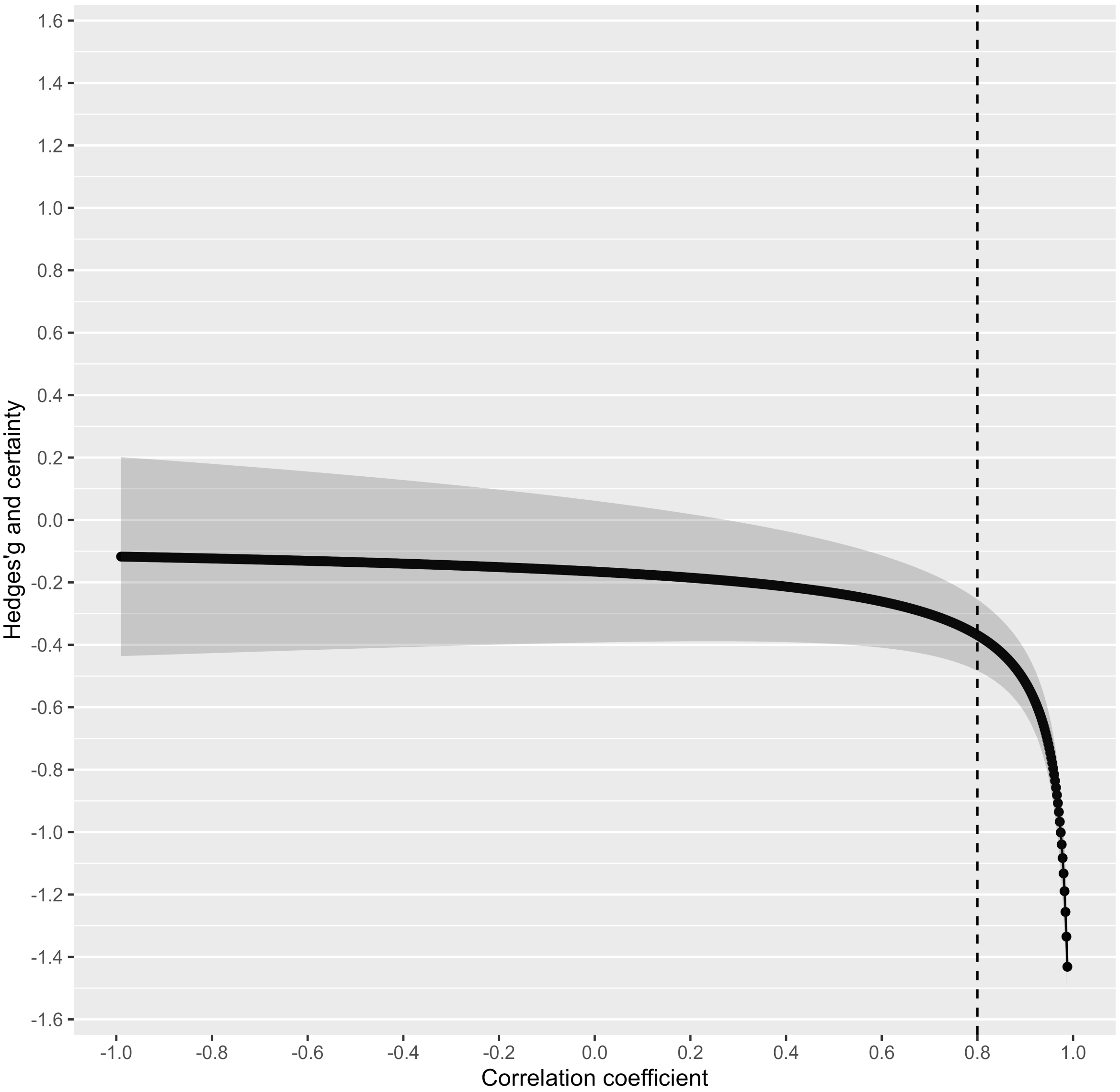

Figure 1 | Specification plot of the sample-size weighted means and confidence intervals of Hedges' g along the grid of correlation coefficients across studies.

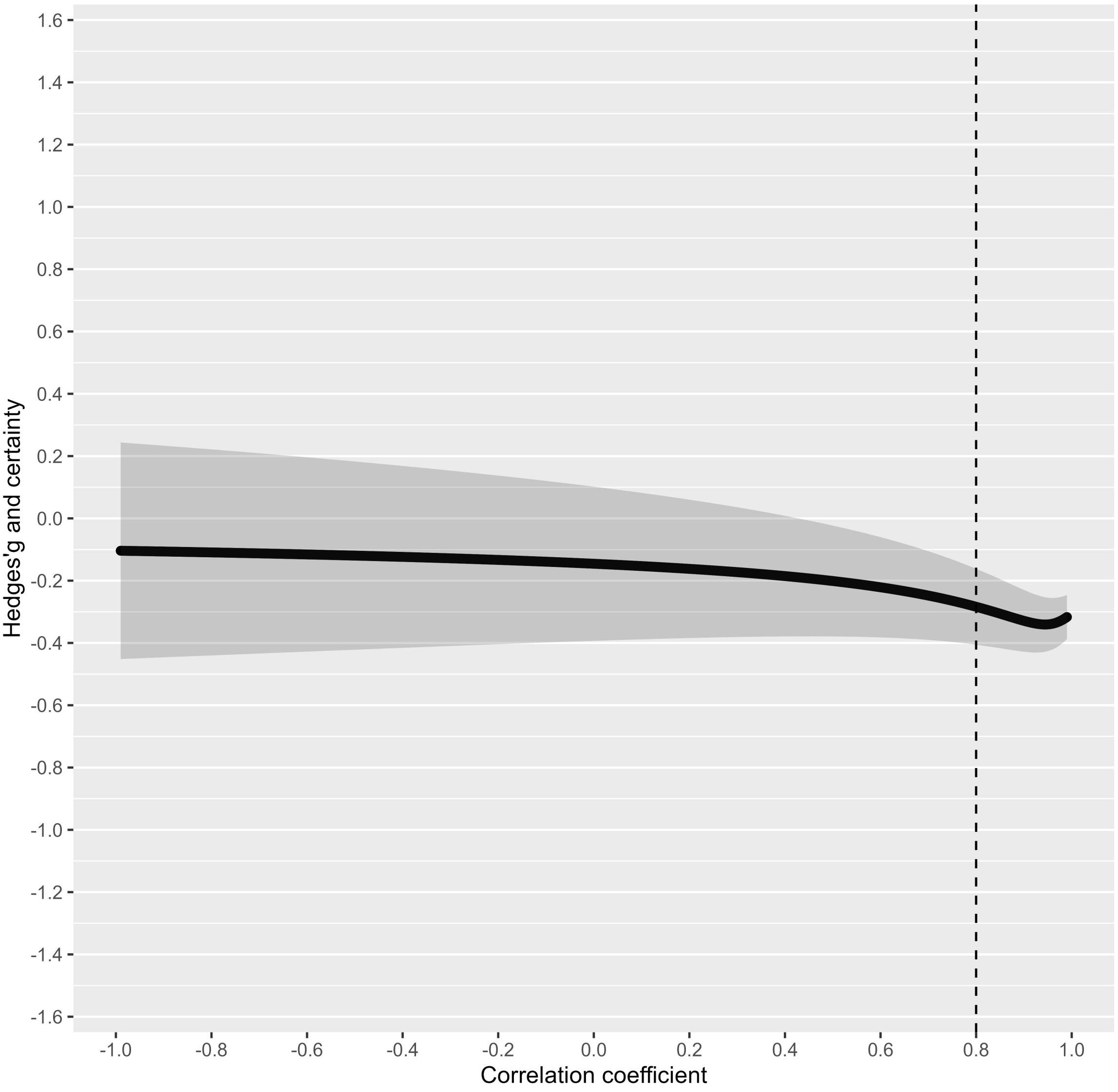

Figure 2 | Specification plot of the inverse variance weighted means and confidence intervals of Hedges' g along the grid of correlation coefficients across studies.
