# Supplementary material for: The effects of waiting time for outpatient psychotherapeutic interventions on patient-reported outcomes in adolescents and adults with eating disorders: a systematic review and meta-analysis
Source: J Eat Disord. 2026 Jun 5;14:129. doi: 10.1186/s40337-026-01660-4 (PMC13248287; doi:10.1186/s40337-026-01660-4)
Supplement: Supplementary file 7 — Additional file 7. Explanation for study exclusions. [file 40337_2026_1660_MOESM7_ESM.pdf]

Additional file 7

Explanation for certain study exclusions

One study technically met our eligibility criteria but was excluded because it differed considerably from our objective and the other included studies.[1] Previous treatment within the last two years was required to ensure participants would not be overwhelmed by their eating disorder symptoms when entering the study. While it is likely that some participants in other studies have also received recent treatment, this was not an eligibility criterion. Therefore, these samples were expected to be more representative of the underlying target population.

Reference List

1. Berardi KL. The clinical effectiveness of cognitive behaviour therapy for the treatment of body image disturbance in women with eating disorders. University of Windsor; 2008; https://scholar.uwindsor.ca/etd/8021
