# Supplementary material for: The effects of waiting time for outpatient psychotherapeutic interventions on patient-reported outcomes in adolescents and adults with eating disorders: a systematic review and meta-analysis
Source: J Eat Disord. 2026 Jun 5;14:129. doi: 10.1186/s40337-026-01660-4 (PMC13248287; doi:10.1186/s40337-026-01660-4)
Supplement: Supplementary file 8 — Additional file 8. Exclusion criteria, psychiatric comorbidities and BMI for the WLCGs of included studies. [file 40337_2026_1660_MOESM8_ESM.pdf]

Additional file 8

Table | Exclusion criteria, psychiatric comorbidities and BMI for the WLCGs of included studies.

First author, year	Psychiatric and medical exclusion criteria	Other relevant exclusion criteria	Psychiatric comorbidities (%)	BMI eligibility in kg/m^2	Mean BMI in kg/m^2 (SD)
Arcelus et al., 2012	high levels of suicidality;<br>high levels psychiatric comorbidity	none	NR	all	NR
Berking et al., 2022	high risk of suicide; co-occurring psychotic or bipolar disorders, alcohol/substance dependence within the past six months; borderline personality disorder; medical disorders affecting weight or ability to participate	current participation in psychotherapeutic treatment other than those offered in the study; current participation in a weight control program; weight-affecting medication; pregnancy	lifetime major depression (35.00)<br>lifetime social phobia (5.00)<br>lifetime alcohol abuse (0.00)<br>current major depression (2.50)<br>current specific phobia (2.50)	BMI < 45	NR
Fairburn et al., 2009	coexisting Axis I psychiatric disorders precluding ED-focused treatment; medical instability	prior receipt of a treatment closely resembling enhanced cognitive-behavioural therapy or an evidence-based treatment for the same ED; psychiatric treatment (patients were weaned off it before entering the study), except for clinically warranted stable antidepressant medication; pregnancy	major depressive episode (21.40)<br>any anxiety disorder (21.40)<br>substance abuse (21.70)[1]	BMI > 17.5	22.90 (3.92)
Glisenti et al., 2021	AN or BN; high suicide risk; current psychosis, intellectual disability; drug or alcohol abuse	current treatment for obesity; pregnancy	NR	all	NR
Krohmer et al., 2022	severe suicidal ideation; current presence of paranoid symptoms; current manic episode; borderline personality disorder; current alcohol or substance-related dependence; acute or chronic illness affecting weight	ongoing therapy; current pregnancy or lactation	NR	all	33.47 (6.42)
Lewer et al., 2017	suicidal tendencies; deliberate self-harm behaviour; personality disorders	current psychotherapy; current intake of psychotropic drugs; male sex; pregnancy	major depression (52.63)<br>social phobia (10.52)<br>panic disorder (5.26)<br>PTSD (5.26)	BMI > 25	36.80 (5.08)
Masson et al., 2013	active psychosis	current psychotherapy for binge eating; use of compensatory behaviours at least once a week over the past three months; unstable dose of psychotropic medication over the last three months	NR	BMI > 17.5	38.83 (8.86)
Schlup et al., 2009	severe Diagnostic and Statistical Manual of Mental Disorders-IV-TR disorders warranting immediate treatment, (e.g. major depression with acute suicidal risk, psychosis, bipolar disorder, or current substance use disorder)	participation in a diet program or another psychotherapy; treatment with weight loss medication (current or during the past 3 months), or previous surgical treatment of obesity; male sex; pregnancy	lifetime depression (22.20)<br>lifetime anxiety (11.10)<br>current depression (11.10)<br>current anxiety (11.10)<br>comorbid axis II disorder (11.10)	all	34.30 (9.10)
Wagner et al., 2016	current AN or BN; severe major depressive symptoms or acute suicidal ideation; history of psychotic or	ongoing psychotherapy; bariatric surgery; pregnancy	NR	all	31.80 (8.10)

First author, year	Psychiatric and medical exclusion criteria	Other relevant exclusion criteria	Psychiatric comorbidities (%)	BMI eligibility in kg/m^2	Mean BMI in kg/m^2 (SD)
	dissociative symptoms; any severe substance abuse or dependence disorder; any serious medical conditions influencing weight or eating				

[1] Assessed with an extension of the Eating Disorder Examination interview, no formal diagnosis of a substance use disorder.

Note: AN = anorexia nervosa; BMI = body mass index; BN = bulimia nervosa; ED = eating disorder; NR = not reported; PTSD = post-traumatic stress disorder; SD = standard deviation.
