# Supplementary material for: The effects of waiting time for outpatient psychotherapeutic interventions on patient-reported outcomes in adolescents and adults with eating disorders: a systematic review and meta-analysis
Source: J Eat Disord. 2026 Jun 5;14:129. doi: 10.1186/s40337-026-01660-4 (PMC13248287; doi:10.1186/s40337-026-01660-4)
Supplement: Supplementary file 9 — Additional file 9. PROGRESS equity characteristics for the WLCGs of included studies. [file 40337_2026_1660_MOESM9_ESM.pdf]

Additional file 9

Table | PROGRESS equity characteristics for the WLCGs of included studies.

First author, year	Place of Residence	Race/ethnicity/ nationality/ language (%)	Occupation/ employment (%)	Religion	Education (%)	SES/ income (%)	Social capital (%)
Arcelus et al., 2012	Leicester area[1], United Kingdom	NR	NR	NR	NR	NR	NR
Berking et al., 2022	Germany	German speaking (100.00)	NR	NR	middle school (15.79)<br>secondary school (42.10)<br>university (39.47)<br>other (2.63)	NR	single (21.05)<br>relationship/married (68.42)<br>separated/divorced (10.53)<br>widowed (0.00)
Fairburn et al., 2009	Central Oxfordshire and Leicester city, United Kingdom	White (94.10)<br>Asian (2.00)<br>Mixed (3.90)	higher (25.50)<br>intermediate (11.80)<br>lower (11.80)<br>unclassifiable (17.60)<br>full-time student (33.30)	NR	NR	NR	single/never married (78.4)<br>married/living as such (17.6)<br>separated/divorced (3.9)
Glisenti et al., 2021	Queensland area[1], Australia	English speaking (100.00)	full-time job (60.00)<br>part-time job (40.00)<br>keeping house (0.00)<br>school/training (0.00)<br>disabled (0.00)	NR	grades 7-12 (20.00)<br>high school or equivalent (10.00)<br>part college/trade school (10.00)<br>2-year college/trade school (30.00)<br>4-year college (30.00)<br>part graduate school (0.00)	NR	married/with someone (50.00)<br>widowed (0.00)<br>divorced/annulled (10.00)<br>separated (20.00)<br>never married (20.00)
Krohmer et al., 2022	Tübingen area[1], Germany	German (93.10)<br>other nationality (6.90)	NR	NR	≥13 years of school (72.41)<br>>9 and <13 years of school (24.12)<br>≤9 years of school (3.45)	≥3,000 €/mth. (32.43)<br>>1,500 and <3,000 €/mth. (48.28)<br>≤1,500 €/mth. (10.35)	NR
Lewer et al., 2017	Bochum area[1], Germany	NR	NR	NR	NR	NR	NR
Masson et al., 2013	Calgary, Canada	Asian (3.33)<br>Black/African (3.33)<br>Caucasian/European (90.00)<br>Multiracial (3.32)	unemployed (10.00)<br>employed part-time (10.00)<br>employed full-time (70.00)<br>retired (10.00)	NR	mean years of education (SD) 15.17 (3.21)	NR	single (33.33)<br>married (33.33)<br>common-law (10.00)<br>divorced (23.33)<br>widowed (0.00)
Schlup et al., 2009	Basel area[1], Switzerland	NR	NR	NR	NR	NR	NR
Wagner et al., 2016	Germany, Austria, Switzerland	NR	NR	NR	low (10.00)<br>medium (20.00)<br>high (70.00)	NR	married/cohabiting (57.10)<br>children (47.10)

[1] Refers to the area of treatment centre and not necessarily to the actual place of residence of the participants.

Note: Mth. = month; NR = not reported; PROGRESS = place of residence, race, occupation, gender, religion, education, socioeconomic status, and social capital[1]; SES = socioeconomic status.

Reference List

1. O'Neill J, Tabish H, Welch V, Petticrew M, Pottie K, Clarke M, et al. Applying an equity lens to interventions: using PROGRESS ensures consideration of socially stratifying factors to illuminate inequities in health. Journal of Clinical Epidemiology. 2014;67:56–64. https://doi.org/10.1016/j.jclinepi.2013.08.005
