# Supplementary material for: The effects of waiting time for outpatient psychotherapeutic interventions on patient-reported outcomes in adolescents and adults with eating disorders: a systematic review and meta-analysis
Source: J Eat Disord. 2026 Jun 5;14:129. doi: 10.1186/s40337-026-01660-4 (PMC13248287; doi:10.1186/s40337-026-01660-4)
Supplement: Supplementary file 10 — Additional file 10. Tables of Risk of bias assessment. [file 40337_2026_1660_MOESM10_ESM.pdf]

Additional file 10

Table 1| Risk of bias assessment with justifications for each judgement in randomised trials assessed with RoB 2.

First author, year	D1	D2	D3	D4	D5	Overall
Berking et al., 2022	**1.1/1.2** N/Y, initially block randomisation was performed but then the authors switched to a procedure where the next eligible patient was allocated to the IG until the minimum group size of four was reached, the authors report "allocation depended exclusively on the rank of the participant in the recruitment order and was therefore as random as it would have been if the combination of the rank in the recruitment order and the block-randomized indicator of study condition had been the basis for the allocation"; assessors were blind to this decision and the decision was made prior to the baseline assessment<br>**1.3** N, "The conditions did not differ significantly regarding sociodemographics, comorbidity and baseline levels of any of the outcome variables.", also see Table 1<br>**Some concerns**	**2.1/2.2** Y/Y, blinding was not possible because of the nature of the IG and WLCG<br>**2.3** NI, no deviations reported<br>**2.4** NA<br>**2.5** NA<br>**2.6** Y, intention-to-treat<br>**2.7** NA<br>**Some concerns**	**3.1** N, 87% of study completers in the IG and 83% in the WLCG<br>**3.2** PY, "We conducted complementary completer analyses for all outcomes, which differed only slightly in p-values but led to the same conclusions regarding changes in dependent variables (…)"<br>**3.3/3.4** NA/NA<br>**Low**	**4.1** N, EDE-Q was used, which was our pre-defined outcome measure, and in accordance with the ICHOM recommendations<br>**4.2** N, same PROM was used, i.e. the EDE-Q<br>**4.3** Y, due to the nature of a PROM, the participants were the assessors and knew whether or not they were currently receiving the intervention<br>**4.4/4.5** Y/Y, the knowledge of receiving treatment can lead to hope and optimism, while being on the waitlist can lead to frustration and the belief that nothing will change; however, being on the waitlist for a study also means that treatment is likely to be available soon, which can also lead to hope; an influence on outcome assessment is likely<br>**High**	**5.1** NI, no pre-specified analysis plan found<br>**5.2** N, EDE-Q scores can only be measured using the EDE-Q; time points were defined a priori<br>**5.3** NI, analysis intentions are not available<br>**Some concerns**	**High**
Fairburn et al., 2009	**1.1/1.2** Y/Y, "A computer-based minimization algorithm was used by one of the authors (H.A.D., who had no involvement in recruitment) to allocate patients to the four treatment conditions, balancing gender, eating disorder	**2.1/2.2** Y/Y, blinding was not possible because of the nature of the IG and WLCG<br>**2.3** NI, no deviations reported<br>**2.4** NA<br>**2.5** NA	**3.1** N, drop-out > 5%, see Figure 1<br>**3.2** PN, "(…) the analyses were by intent-to-treat with the initial data brought forward. Other imputation methods were tested, but as there were few	**4.1** N, EDE-Q was used, which was our pre-defined outcome measure, and in accordance with the ICHOM recommendations<br>**4.2** N, same PROM was used, i.e. the EDE-Q	**5.1** NI, no pre-specified analysis plan found<br>**5.2** N, EDE-Q scores can only be measured using the EDE-Q; time points were defined a priori	**High**

First author, year	D1	D2	D3	D4	D5	Overall
	diagnosis, BMI, and need to remain on psychotropic medication. When groups were evenly balanced, pre-prepared blocked randomization lists of varying size were used to allocate patients to the four conditions."<br><br>**1.3** N, "The study groups were balanced on all baseline factors except that patients assigned to immediate CBT-Eb were less likely to have a current major depressive episode or a history of anorexia nervosa (Table 1). Adjusting for these two factors in the analyses made no difference to the findings."<br><br>**Low**	**2.6** Y, intention-to-treat<br><br>**2.7** NA<br><br>**Some concerns**	missing data, this made little difference to the main findings."<br><br>**3.3/3.4** Y/PY, likely to depend on the health status of the participant; reasons for missing outcome data not reported<br><br>**High**	**4.3** Y, due to the nature of a PROM, the participants were the assessors and knew whether or not they were currently receiving the intervention<br><br>**4.4/4.5** Y/Y, the knowledge of receiving treatment can lead to hope and optimism, while being on the waitlist can lead to frustration and the belief that nothing will change; however, being on the waitlist for a study also means that treatment is likely to be available soon, which can also lead to hope; an influence on outcome assessment is likely<br><br>**High**	**5.3** NI, analysis intentions are not available<br><br>**Some concerns**	
Glisenti et al., 2021	**1.1/1.2** Y/Y, "(…) participants were randomly allocated to either an immediate EFT intervention or 12-week EFT wait-list using a block randomisation method"<br><br>**1.3** N, "No significant demographic differences were found between the immediate EFT intervention and EFT wait-list control groups in relation to mean age (years), mean age at first binge (years), gender, marital status, education, and employment status. See Table 3."<br><br>**Low**	**2.1/2.2** Y/Y, blinding was not possible because of the nature of the IG and WLCG<br><br>**2.3** NI, no deviations reported<br><br>**2.4** NA<br><br>**2.5** NA<br><br>**2.6** PN, most likely per protocol<br><br>**2.7** NI<br><br>**High**	**3.1** NI, drop-out < 5%, see Figure 1; however, no information on missing data reported<br><br>**3.2** PN, "Missing data were managed using pairwise deletion."<br><br>**3.3/3.4** Y/PY, likely to depend on the health status of the participant; reasons for missing outcome data not reported<br><br>**High**	**4.1** N, EDE-Q was used, which was our pre-defined outcome measure, and in accordance with the ICHOM recommendations<br><br>**4.2** N, same PROM was used, i.e. the EDE-Q<br><br>**4.3** Y, due to the nature of a PROM, the participants were the assessors and knew whether or not they were currently receiving the intervention<br><br>**4.4/4.5** Y/Y, the knowledge of receiving treatment can lead to hope and optimism, while being on the waitlist can lead to frustration and the belief that nothing will change; however, being on the waitlist for a study also means that treatment is	**5.1** NI, no pre-specified analysis plan found<br><br>**5.2** PN, only OBEs and OBEDs reported but this decision seems to be aligned with the objective rather than being the result of outcome selection<br><br>**5.3** NI, analysis intentions are not available<br><br>**Some concerns**	**High**

First author, year	D1	D2	D3	D4	D5	Overall
				likely to be available soon, which can also lead to hope; an influence on outcome assessment is likely<br><br>**High**		
Krohmer et al., 2022	**1.1/1.2** Y/NI, "In the present study, women with BED were randomly assigned to either repeated mirror exposure (intervention group, IG) or a waiting list control group (WL)."; concealment not reported<br><br>**1.3** N, "At baseline, groups did not differ in age, weight, self-reported eating pathology, body image concerns, or severity of depression.", also see Table 1<br><br>**Some concerns**	**2.1/2.2** Y/Y, blinding was not possible because of the nature of the IG and WLCG<br><br>**2.3** NI, no deviations reported<br><br>**2.4** NA<br><br>**2.5** NA<br><br>**2.6** Y, intention-to-treat<br><br>**2.7** NA<br><br>**Some concerns**	**3.1** N, drop-out rate was 23.5%<br><br>**3.2** PN, "Multiple imputation approach was applied to deal with missing data in the ITT analysis."<br><br>**3.3/3.4** Y/PY, likely to depend on the health status of the participant; reasons for missing outcome data not reported<br><br>**High**	**4.1** N, EDE-Q was used, which was our pre-defined outcome measure, and in accordance with the ICHOM recommendations<br><br>**4.2** N, same PROM was used, i.e. the EDE-Q<br><br>**4.3** Y, due to the nature of a PROM, the participants were the assessors and knew whether or not they were currently receiving the intervention<br><br>**4.4/4.5** Y/Y, the knowledge of receiving treatment can lead to hope and optimism, while being on the waitlist can lead to frustration and the belief that nothing will change; however, being on the waitlist for a study also means that treatment is likely to be available soon, which can also lead to hope; an influence on outcome assessment is likely<br><br>**High**	**5.1** N, "ANCOVA was used as recommended by the reviewers. Originally, the analysis was planned with ANOVAs."<br><br>**5.2** PN, not all subscales reported, but this decision seems to be aligned with the objective rather than being the result of outcome selection<br><br>**5.3** NI, analysis intentions are not available<br><br>**Some concerns**	**High**
Lewer et al., 2017	**1.1/1.2** Y/NI, "An unrestricted randomization procedure was used. By casting a dice, participants were then randomized either to the IG or the CG."; concealment not reported	**2.1/2.2** Y/Y, blinding was not possible because of the nature of the IG and WLCG<br><br>**2.3** NI, no deviations reported<br><br>**2.4** NA<br><br>**2.5** NA	**3.1** NI, drop-out < 5%; however, no information on missing data reported<br><br>**3.2** N, no corresponding methods described<br><br>**3.3/3.4** Y/PY, likely to depend on the health status of the	**4.1** N, EDE-Q was used, which was our pre-defined outcome measure, and in accordance with the ICHOM recommendations<br><br>**4.2** N, same PROM was used, i.e. the EDE-Q	**5.1** NI, no pre-specified analysis plan found<br><br>**5.2** N, EDE-Q scores can only be measured using the EDE-Q; time points were defined a priori	**High**

First author, year	D1	D2	D3	D4	D5	Overall
	**1.3** NI, no baseline characteristics provided<br>**Some concerns**	**2.6** N, completer-analysis<br>**2.7** NI<br>**High**	participant; reasons for missing outcome data not reported<br>**High**	**4.3** Y, due to the nature of a PROM, the participants were the assessors and knew whether or not they were currently receiving the intervention<br>**4.4/4.5** Y/Y, the knowledge of receiving treatment can lead to hope and optimism, while being on the waitlist can lead to frustration and the belief that nothing will change; however, being on the waitlist for a study also means that treatment is likely to be available soon, which can also lead to hope; an influence on outcome assessment is likely<br>**High**	**5.3** NI, analysis intentions are not available<br>**Some concerns**	
Masson et al., 2013	**1.1/1.2** Y/Y, "(…) randomized to either the treatment or wait-list condition by another researcher (PCM) using an urn randomization program that stratified randomization based on age (age under 35 years versus age 35 years and older) and gender to help ensure equal distribution of age and gender among the groups despite the small sample size. (…) The assessor was blind to group assignment for baseline and post-treatment assessments."<br>**1.3** N, "Participants in the treatment and control groups did not differ in binge eating frequency, compensatory behaviour use, BMI, age, gender, ethnicity, employment status, years of education, or	**2.1/2.2** Y/Y, blinding was not possible because of the nature of the IG and WLCG<br>**2.3** NI, no deviations reported<br>**2.4** NA<br>**2.5** NA<br>**2.6** Y, intention-to-treat<br>**2.7** NA<br>**Some concerns**	**3.1** N, drop-out 30% in IG, 10% in WLCG<br>**3.2** N, "The last observation carried forward procedure was used in all analyses to account for missing data."<br>**3.3/3.4** Y/PY, likely to depend on the health status of the participant; "(…) individuals who dropped out were younger than those who completed treatment."<br>**High**	**4.1** N, EDE-Q was used, which was our pre-defined outcome measure, and in accordance with the ICHOM recommendations<br>**4.2** N, same PROM was used, i.e. the EDE-Q<br>**4.3** Y, due to the nature of a PROM, the participants were the assessors and knew whether or not they were currently receiving the intervention<br>**4.4/4.5** Y/Y, the knowledge of receiving treatment can lead to hope and optimism, while being on the waitlist can lead to frustration and the belief that nothing will change; however, being on the waitlist for a study also means that treatment is	**5.1** NI, no pre-specified analysis plan found<br>**5.2** N, EDE-Q scores can only be measured using the EDE-Q; time points were defined a priori<br>**5.3** NI, analysis intentions are not available<br>**Some concerns**	**High**

First author, year	D1	D2	D3	D4	D5	Overall
	marital status.", also see Tables 2 and 3<br><br>**Low**			likely to be available soon, which can also lead to hope; an influence on outcome assessment is likely<br><br>**High**		
Schlup et al., 2009	**1.1/1.2** Y/NI, "(…) were randomly assigned to either the immediate treatment or the waitlist condition using a permuted block design."; concealment not reported<br><br>**1.3** PN, see Table 1; we assume differences to be due to the small sample sizes per group<br><br>**Some concerns**	**2.1/2.2** Y/Y, blinding was not possible because of the nature of the IG and WLCG<br><br>**2.3** NI, no deviations reported<br><br>**2.4** NA<br><br>**2.5** NA<br><br>**2.6** PY, information not explicitly reported, but based on Table 2 and Table 3, we assume intention-to-treat<br><br>**2.7** NI<br><br>**Some concerns**	**3.1** N, drop-out rate of 13%<br><br>**3.2** PY, "To analyze continuously distributed outcome measures, we used linear mixed models (…) which were shown to lead to more efficient and less biased results compared with complete case analyses or analyses in which missing values have been imputed prior to the analysis using the last observation carried forward method (…)"<br><br>**3.3/3.4** NA/NA<br><br>**Low**	**4.1** N, EDE-Q was used, which was our pre-defined outcome measure, and in accordance with the ICHOM recommendations<br><br>**4.2** N, same PROM was used, i.e. the EDE-Q<br><br>**4.3** Y, due to the nature of a PROM, the participants were the assessors and knew whether or not they were currently receiving the intervention<br><br>**4.4/4.5** Y/Y, the knowledge of receiving treatment can lead to hope and optimism, while being on the waitlist can lead to frustration and the belief that nothing will change; however, being on the waitlist for a study also means that treatment is likely to be available soon, which can also lead to hope; an influence on outcome assessment is likely<br><br>**High**	**5.1** NI, no pre-specified analysis plan found<br><br>**5.2** N, EDE-Q scores can only be measured using the EDE-Q; time points were defined a priori<br><br>**5.3** NI, analysis intentions are not available<br><br>**Some concerns**	**High**
Wagner et al., 2016	**1.1/1.2** Y/Y, "The computer-assisted randomization procedure was conducted by the Center of Clinical Trials (University of Leipzig) and stratification by the number of OBEs was implemented."	**2.1/2.2** Y/Y, blinding was not possible because of the nature of the IG and WLCG<br><br>**2.3** NI, no deviations reported<br><br>**2.4** NA<br><br>**2.5** NA	**3.1** N, "In the TG, 19 (27.5%) participants dropped out between pre- and posttreatment assessment. In the WL, 6 patients (8.6%) did not complete the post-waiting assessment (see Figure 1)."	**4.1** N, EDE-Q was used, which was our pre-defined outcome measure, and in accordance with the ICHOM recommendations<br><br>**4.2** N, same PROM was used, i.e. the EDE-Q	**5.1** NI, no pre-specified analysis plan found<br><br>**5.2** N, EDE-Q scores can only be measured using the EDE-Q; time points were defined a priori	**High**

First author, year	D1	D2	D3	D4	D5	Overall
	**1.3** N, "The TG and the WL did not differ in any of the demographic variables at baseline or with regard to binge-eating frequency, other eating disorder symptoms, depression, anxiety, and body weight or BMI at baseline (see Table 1)."<br>**Low**	**2.6** Y, intention-to-treat<br>**2.7** NA<br>**Some concerns**	**3.2** N, "(…) missing data at posttreatment and follow-up assessments were imputed using the “last observation carried forward” (LOCF) method."<br>**3.3/3.4** Y/PY, likely to depend on the health status of the participant; reasons for missing outcome data not reported<br>**High**	**4.3** Y, due to the nature of a PROM, the participants were the assessors and knew whether or not they were currently receiving the intervention<br>**4.4/4.5** Y/Y, the knowledge of receiving treatment can lead to hope and optimism, while being on the waitlist can lead to frustration and the belief that nothing will change; however, being on the waitlist for a study also means that treatment is likely to be available soon, which can also lead to hope; an influence on outcome assessment is likely<br>**High**	**5.3** NI, analysis intentions are not available<br>**Some concerns**	

Note: Domains: D1 = Bias arising from the randomisation process; D = 2 Bias due to deviations from intended intervention; D3 = Bias due to missing outcome data; D4 = Bias in measurement of the outcome; D5 = Bias in selection of the reported result. EDE-Q = Eating Disorder Examination-Questionnaire; ICHOM = International Consortium for Health Outcomes Measurement; IG = intervention group; N = no; NA = not applicable; NI = no information; OBE = objective binge episode; OBED = objective binge episode day; PN = probably no; PROM = patient-reported outcome measure; PY = probably yes; WLCG = waitlist control group; Y = yes. For items see Sterne et al.[1].

Table 2 | Risk of bias assessment with justifications for each judgement in the non-randomised study assessed with ROBINS-I.

First author, year	D1[1]	D2	D3	D4	D5	D6	D7	Overall
Arcelus et al., 2012	**1.1** SN; matching was only performed for gender and diagnosis, not for other factors such as age, ED severity and comorbidities<br>**1.2** NA<br>**1.3** NA<br>**1.4** PN; uncontrolled confounding was probably not present<br>**Serious**	**2.1** N; for the WLCG and the IPT-BN 16 group retrospective data were used; no classical allocation was performed<br>**2.2** NA<br>**2.3** Y; all information was recorded before the time the intervention started<br>**2.4** N; not applicable<br>**2.5** Y; all participants were correctly classified because of the use of retrospective data<br>**Low**	**3.1** N; see 2.1<br>**3.2** NA<br>**3.3** Y; participants were followed up from the start of the intervention/waiting period<br>**3.4** NA<br>**3.5** N; selection was based only on characteristics (i.e. the inclusion criteria) observed before the start of intervention<br>**3.6** NA<br>**3.7** NA<br>**3.8** NA<br>**3.9** NA<br>**3.10** NA<br>**Low**	**4.1** N; regular patients at an outpatient treatment centre<br>**4.2** NA<br>**4.3** NA<br>**4.4** NA<br>**4.5** Y; no deviations from assigned intervention strategy<br>**Low**	**5.1** Y; complete data on intervention status were available for all participants<br>**5.2** NI; no information about the extent of missing data provided<br>**5.3** NI; no information about the extent of missing data provided<br>**5.4** NI; no complete case analysis described<br>**5.5** NI; no information, as it is unsure whether a complete case analysis was conducted<br>**5.6** SN; missing data could be due to higher ED severity<br>**5.7** NA<br>**5.8** NA<br>**5.9** NA<br>**5.10** NA<br>**5.11** PN; handling of missing data not described<br>**Critical**	**6.1** N; same PROM was used, i.e. the EDE-Q<br>**6.2** Y; due to the nature of a PROM, the participants were the assessors and knew whether or not they were currently receiving the intervention<br>**6.3** SY; the knowledge of receiving treatment can lead to hope and optimism, while being on the waitlist can lead to frustration and the belief that nothing will change; however, being on the waitlist for a study also means that treatment is likely to be available soon, which can also lead to hope; an influence on outcome assessment is likely<br>**Serious**	**7.1** NI; no pre-determined analysis plan found<br>**7.2** N; there is only one possible way to assess the EDE-Q Global Score and subscales<br>**7.3** PN; EDE-Q Global Score and subscales were reported as means with standard deviations or mean differences<br>**7.4** NI; analysis intentions not available<br>**Moderate**	**Critical**

[1] Variant A was used.

Note: Domains: D1 = Bias due to confounding; D2 = Bias due to selection of participants; D3 = Bias in classification of interventions; D4 = Bias due to deviations from intended interventions; D5 = Bias due to missing data; D6 = Bias in measurement of outcomes; D7 = Bias in selection of the reported result. ED = eating disorder; EDE-Q = Eating Disorder Examination-Questionnaire; IPT-BN 16 = conventional Interpersonal Psychotherapy for Bulimia Nervosa (16-20 sessions); N = no; NA = not applicable; NI = no information; PN = probably no; PROM = patient-reported outcome measure; PY = probably yes; SN = strong no; Y = yes. For items see Sterne et al.[2].

Reference List

1. Sterne JAC, Savović J, Page MJ, Elbers RG, Blencowe NS, Boutron I, et al. RoB 2: a revised tool for assessing risk of bias in randomised trials. BMJ. BMJ; 2019;l4898. https://doi.org/10.1136/bmj.l4898

2. Sterne JA, Hernán MA, Reeves BC, Savović J, Berkman ND, Viswanathan M, et al. ROBINS-I: a tool for assessing risk of bias in non-randomised studies of interventions. BMJ. BMJ; 2016;i4919. https://doi.org/10.1136/bmj.i4919
