# Supplementary material for: The effects of waiting time for outpatient psychotherapeutic interventions on patient-reported outcomes in adolescents and adults with eating disorders: a systematic review and meta-analysis
Source: J Eat Disord. 2026 Jun 5;14:129. doi: 10.1186/s40337-026-01660-4 (PMC13248287; doi:10.1186/s40337-026-01660-4)
Supplement: Supplementary file 11 — Additional file 11. EDE-Q Global and subscale scores, objective binge episodes and days for the WLCGs. [file 40337_2026_1660_MOESM11_ESM.pdf]

Additional file 11

Table 1 | EDE-Q Global Scores and subscale scores for the WLCGs of included studies.

First author, year	Global Score			Restraint			Weight Concern			Shape Concern			Eating Concern		
	Pre M (SD)	Post M (SD)	MD (SD)	Pre M (SD)	Post M (SD)	MD (SD)	Pre M (SD)	Post M (SD)	MD (SD)	Pre M (SD)	Post M (SD)	MD (SD)	Pre M (SD)	Post M (SD)	MD (SD)
Arcelus et al., 2012	4.65 (0.60)	4.96[1] (0.62)[2]	0.31 (0.61)[3]	4.14 (1.40)	4.50[1] (1.38)[2]	0.36 (1.39)[3]	5.12 (1.00)	5.70[1] (1.06)[2]	0.58 (1.03)[3]	5.22 (0.70)	5.59[1] (0.71)[2]	0.37 (0.71)[3]	4.13 (1.00)	4.11[1] (1.07)[2]	-0.02 (1.04)[3]
Berking et al., 2022	3.12 (0.97)	2.80 (1.06)	-0.32[4] (1.02)[3]	2.09 (1.33)	2.10 (1.36)	0.01[4] (1.35)[3]	3.78 (1.05)	3.28 (1.19)	-0.50[4] (1.13)[3]	4.10 (1.05)	3.71 (1.26)	-0.39[4] (1.17)[3]	2.51 (1.27)	2.13 (1.37)	-0.38[4] (1.32)[3]
Fairburn et al., 2009	4.08 (0.95)	3.99 (1.01)	-0.09 (0.98)[3]	3.79 (1.24)	3.66 (1.22)	-0.13 (1.23)[3]	4.19 (1.41)	4.14 (1.32)	-0.03 (1.37)[3]	4.76 (1.10)	4.65 (1.03)	-0.07 (1.07)[3]	3.60 (1.21)	3.50 (1.41)	-0.12 (1.32)[3]
Glisenti et al., 2021	N/A	N/A	N/A	N/A	N/A	N/A	N/A	N/A	N/A	N/A	N/A	N/A	N/A	N/A	N/A
Krohmer et al., 2022	3.12 (0.85)	2.94 (0.97)	-0.18[4] (0.92)[3]	N/A	N/A	N/A	3.52 (1.12)	3.42 (1.05)	-0.10[4] (1.09)[3]	4.06 (0.81)	3.76 (0.95)	-0.30[4] (0.89)[3]	N/A	N/A	N/A
Lewer et al., 2017	3.08[5] (N/A)	3.10[6] (N/A)	0.02[4] (N/A)	1.82 (1.03)	1.76 (1.04)	-0.06[4] (1.04)[3]	3.81 (1.04)	3.85 (1.21)	0.04[4] (1.13)[3]	4.43 (1.14)	4.48 (0.97)	0.05[4] (1.07)[3]	2.27 (1.07)	2.30 (1.31)	0.03[4] (1.21)[3]
Masson et al., 2013	4.60 (0.85)	4.36 (1.00)	-0.24[4] (0.93)[3]	3.23 (1.27)	3.21 (1.25)	-0.02[4] (1.26)[3]	5.06 (0.86)	4.99 (1.23)	-0.07[4] (1.09)[3]	5.45 (1.12)	5.50 (1.20)	0.05[4] (1.16)[3]	4.67 (1.38)	3.74 (1.46)	-0.93[4] (1.42)[3]
Schlup et al., 2009	3.16[7] (1.06)[7]	3.04[7] (0.90)[7]	-0.12[4] (0.99)[3]	2.34[7] (1.28)[7]	1.80[7] (1.19)[7]	-0.54[4] (1.24)[3]	3.60[7] (1.03)[7]	3.51[7] (1.04)[7]	-0.09[4] (1.04)[3]	4.06[7] (1.48)[7]	4.09[7] (1.27)[7]	0.03[4] (1.39)[3]	2.65[7] (1.36)[7]	2.75[7] (1.22)[7]	0.10[4] (1.30)[3]
Wagner et al., 2016	3.90 (0.90)	3.70 (0.80)	-0.20[4] (0.85)[3]	2.80 (1.40)	2.60 (1.40)	-0.20[4] (1.40)[3]	4.30 (1.00)	4.20 (0.80)	-0.10[4] (0.92)[3]	4.60 (0.80)	4.50 (0.80)	-0.10[4] (0.80)[3]	3.80 (1.20)	3.40 (1.20)	-0.40[4] (1.20)[3]

[1] Post-waiting means were calculated as $M_{post} = M_{pre} + MD$.
[2] Post-waiting standard deviations were approximated as $SD_{post} = SD_{pre} \times$ mean proportional change in SD from pre- to post-waiting across the remaining included studies (SD_{post} / SD_{pre}).
[3] Standard deviations of within-group change scores were calculated as $SD_{within} = \sqrt{(SD_{pre}^2 + SD_{post}^2 - 2 \times r \times SD_{pre} \times SD_{post})}$ assuming a pre-post correlation of $r = 0.5$; sensitivity analyses were conducted using $r = 0.2$ and $r = 0.8$.
[4] Mean differences were calculated as $MD = M_{post} - M_{pre}$.
[5] Pre-waiting EDE-Q global scores were calculated as the mean of the four subscales: (M_{pre} Restraint + M_{pre} Weight Concern + M_{pre} Shape Concern + M_{pre} Eating Concern) / 4.
[6] Post-waiting EDE-Q global scores were calculated analogously using post-waiting subscale means.
[7] Data were obtained from the study authors upon request.
Note: EDE-Q = Eating Disorder Examination-Questionnaire; M = mean; MD = mean difference; N/A = not available; SD = standard deviation, WLCG = waitlist control group.

Table 2 | EDE-Q objective binge episodes and objective binge episode days over a 7-day reference period for the WLCG of Glisenti et al.[1].

First author, year	Objective Binge Episodes			Objective Binge Episode Days		
	**Pre M (SD)**	**Post M (SD)**	**MD (SD)**	**Pre M (SD)**	**Post M (SD)**	**MD (SD)**
Glisenti et al., 2021	5.10 (3.03)	5.10 (2.84)	0.00[1] (2.94)[2]	4.40 (2.41)	4.70 (2.31)	0.30[1] (2.36)[2]

[1] Mean differences were calculated as MD = $M_{post} - M_{pre}$.
[2] Standard deviations of within-group change scores were calculated as $SD_{within} = \sqrt{(SD_{pre}^2 + SD_{post}^2 - 2 \times r \times SD_{pre} \times SD_{post})}$ assuming a pre-post correlation of $r = 0.5$
Note: M = mean; MD = mean difference; N/A = not available; SD = standard deviation.

Reference List

1. Glisenti K, Strodl E, King R, Greenberg L. The feasibility of emotion-focused therapy for binge-eating disorder: a pilot randomised wait-list control trial. J Eat Disord. 2021;9:2. https://doi.org/10.1186/s40337-020-00358-5
