# Supplementary material for: The effects of waiting time for outpatient psychotherapeutic interventions on patient-reported outcomes in adolescents and adults with eating disorders: a systematic review and meta-analysis
Source: J Eat Disord. 2026 Jun 5;14:129. doi: 10.1186/s40337-026-01660-4 (PMC13248287; doi:10.1186/s40337-026-01660-4)
Supplement: Supplementary file 12 — Additional file 12. Forest plots for subgroup analyses. [file 40337_2026_1660_MOESM12_ESM.pdf]

Additional file 12

Forest plots of subgroup analyses

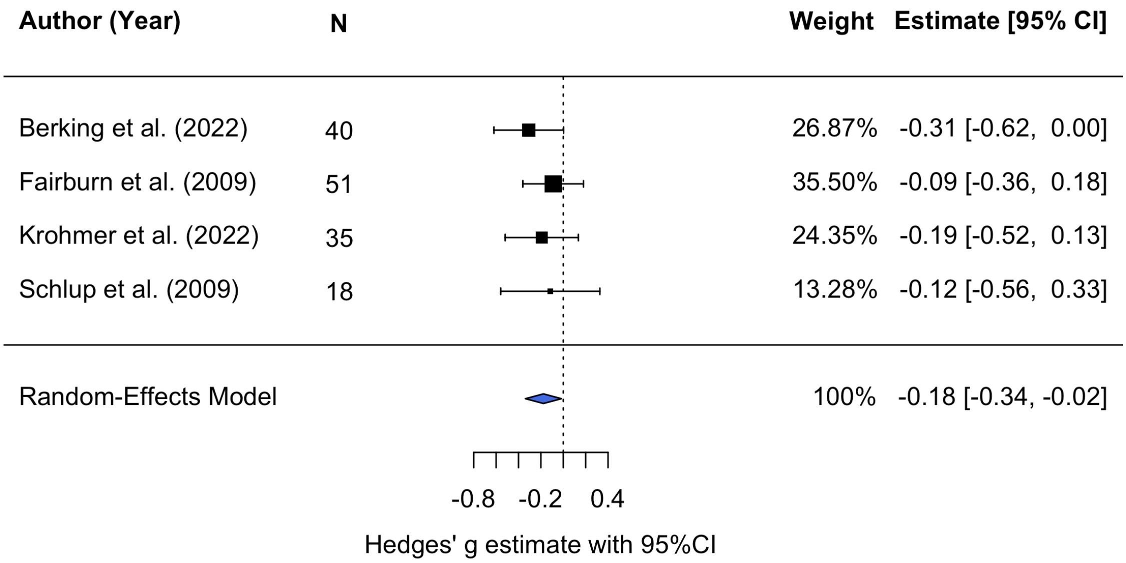

Figure 1 | Forest plot for the subgroup analysis of shorter waiting times (≥10 weeks).

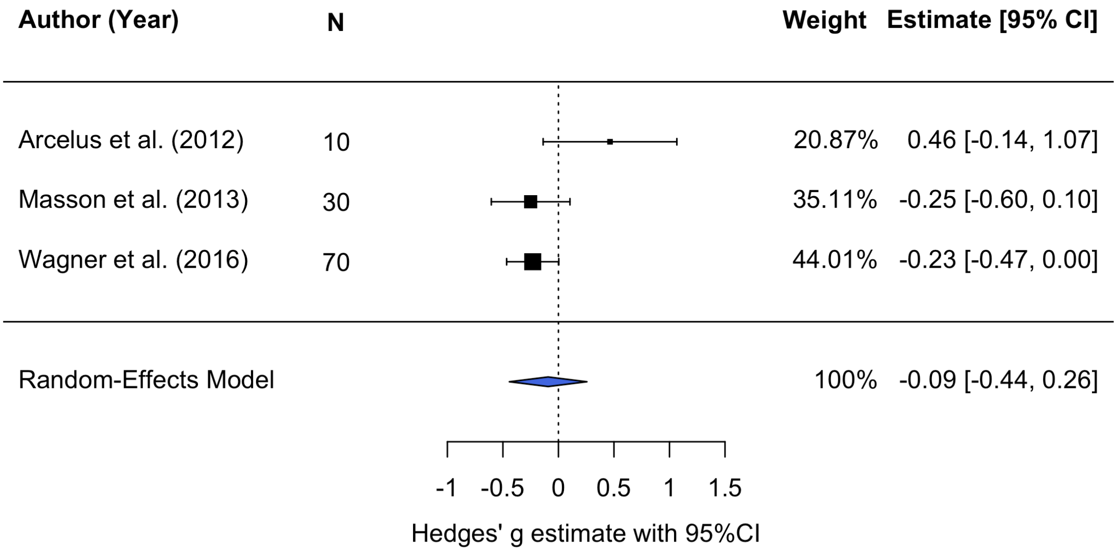

Figure 2 | Forest plot for the subgroup analysis of longer waiting times (<10 weeks).

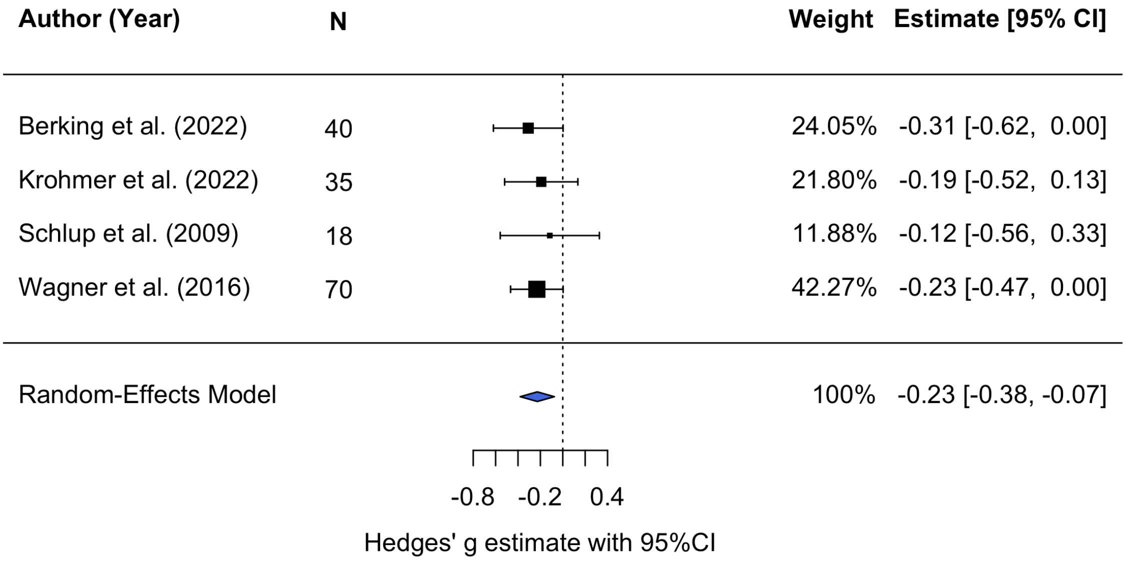

Figure 3 | Forest plot for the subgroup analysis of studies only including BED.

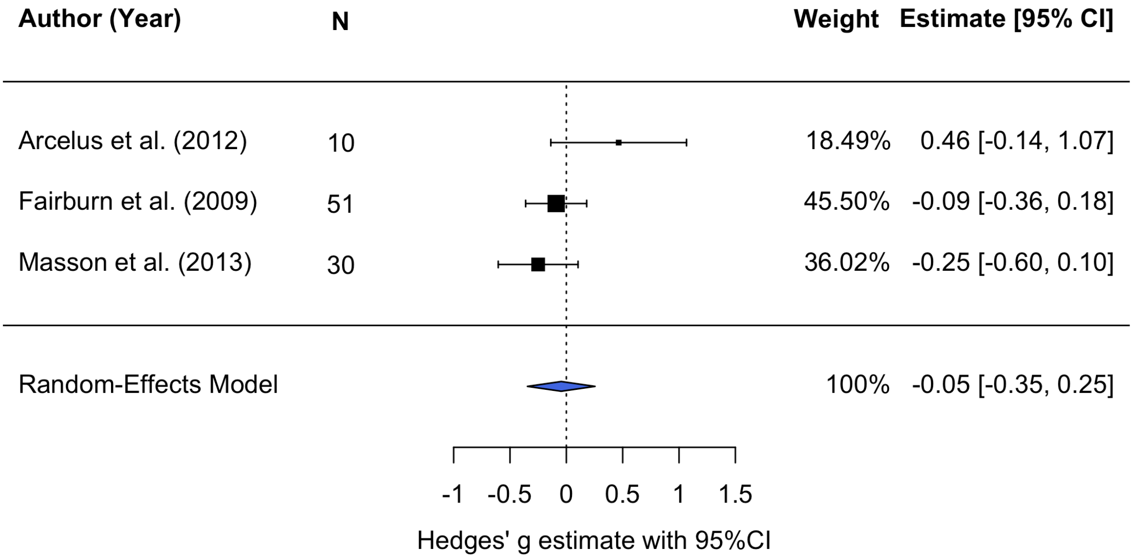

Figure 4 | Forest plot for the subgroup analysis of studies including mixed ED samples (BN, BED, EDNOS).
