# Supplementary material for: The effects of waiting time for outpatient psychotherapeutic interventions on patient-reported outcomes in adolescents and adults with eating disorders: a systematic review and meta-analysis
Source: J Eat Disord. 2026 Jun 5;14:129. doi: 10.1186/s40337-026-01660-4 (PMC13248287; doi:10.1186/s40337-026-01660-4)
Supplement: Supplementary file 13 — Additional file 13. Forest plots for sensitivity analyses. [file 40337_2026_1660_MOESM13_ESM.pdf]

Additional file 13

Forest plots of sensitivity analyses

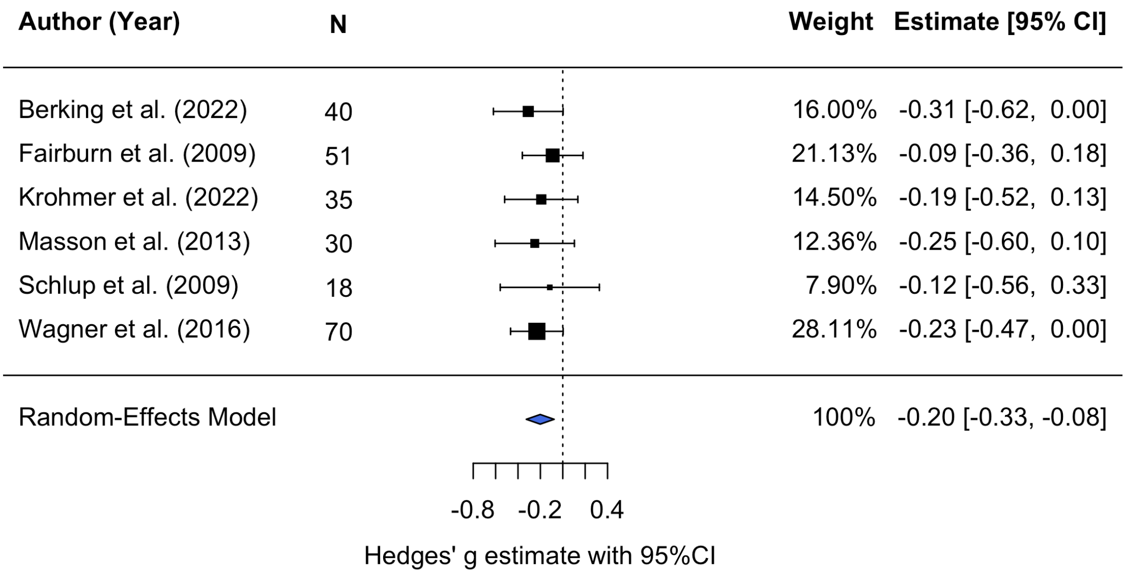

Figure 1 | Forest plot for the sensitivity analysis only including RCTs.

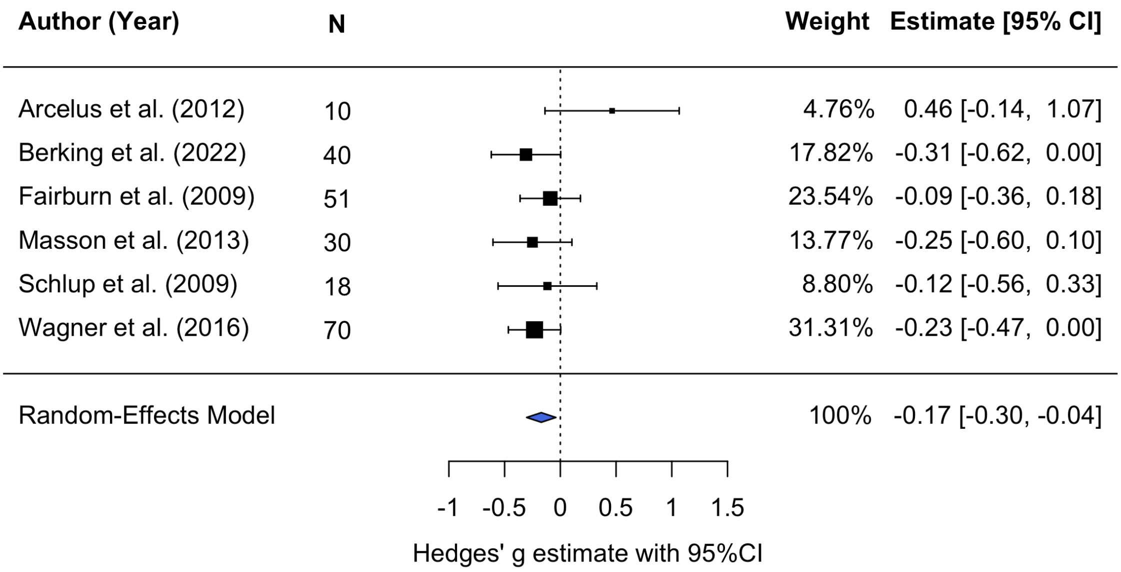

Figure 2 | Forest plot for the sensitivity analysis excluding studies with differing WLCG characteristics.

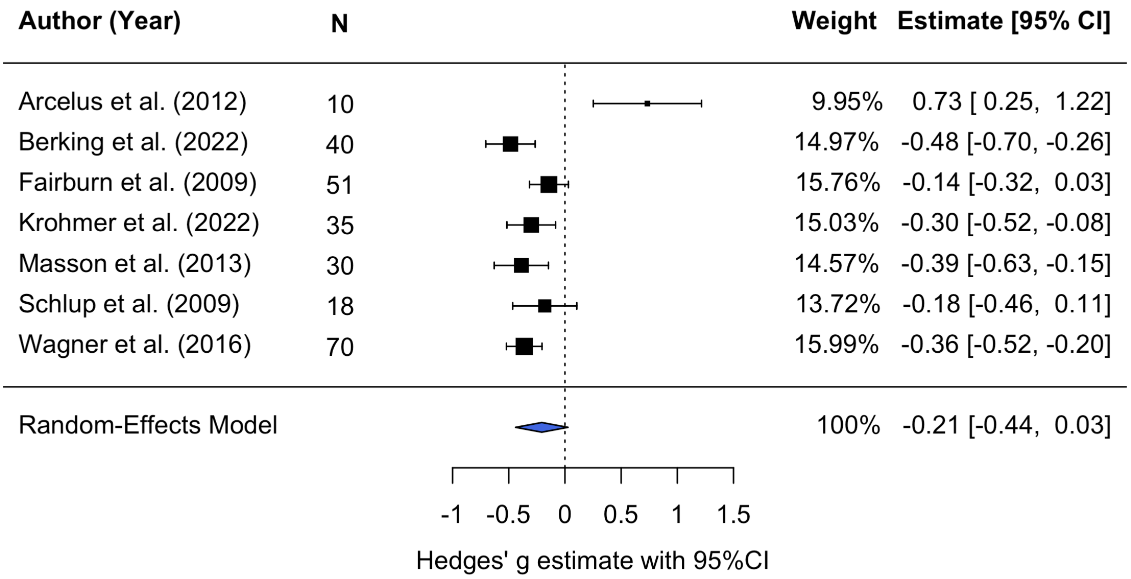

Figure 3 | Forest plot for the sensitivity analysis assuming a pre-post correlation coefficient of r = 0.8.

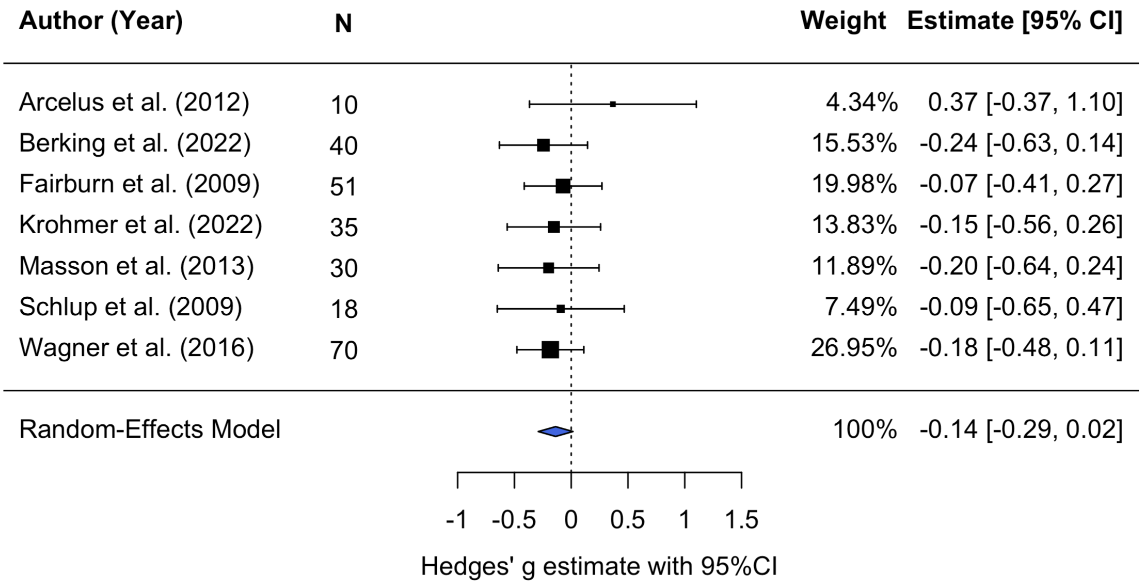

Figure 4 | Forest plot for the sensitivity analysis assuming a pre-post correlation coefficient of r = 0.2.
