# Supplementary material for: The effects of waiting time for outpatient psychotherapeutic interventions on patient-reported outcomes in adolescents and adults with eating disorders: a systematic review and meta-analysis
Source: J Eat Disord. 2026 Jun 5;14:129. doi: 10.1186/s40337-026-01660-4 (PMC13248287; doi:10.1186/s40337-026-01660-4)
Supplement: Supplementary file 14 — Additional file 14. Funnel plots for secondary and sensitivity analyses. [file 40337_2026_1660_MOESM14_ESM.pdf]

Additional file 14

Funnel plots of secondary and sensitivity analyses

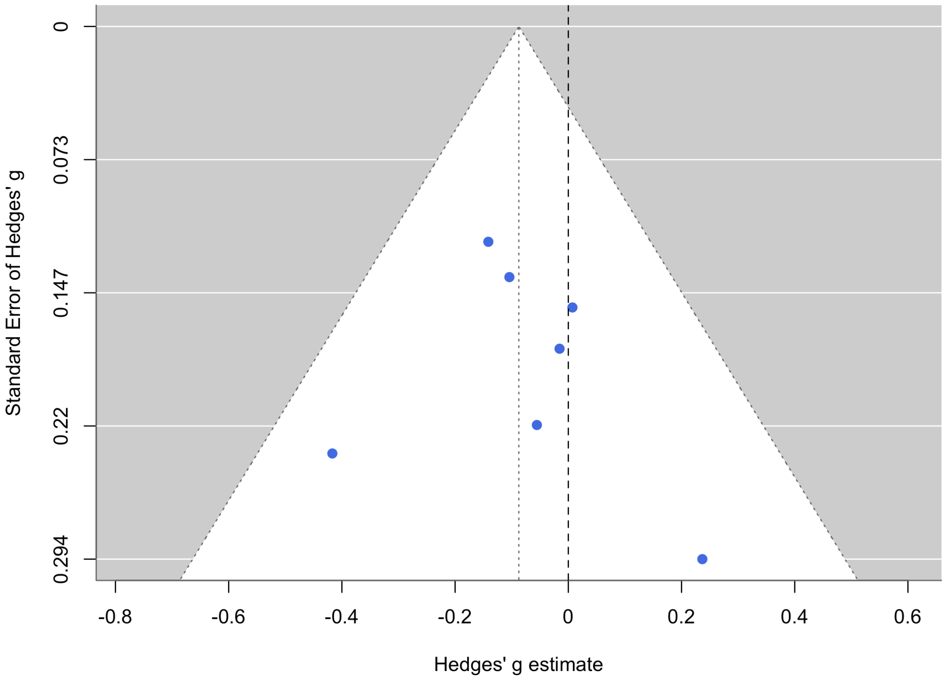

Figure 1 | Funnel plot for the secondary analysis of the EDE-Q subscale Restraint.

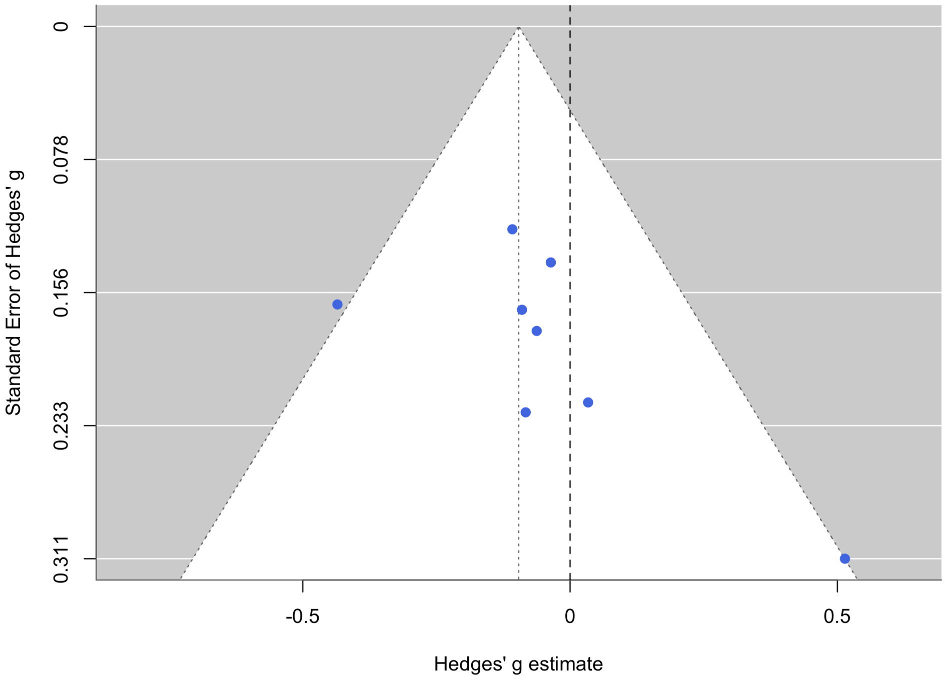

Figure 2 | Funnel plot for the secondary analysis of the EDE-Q subscale Weight Concern.

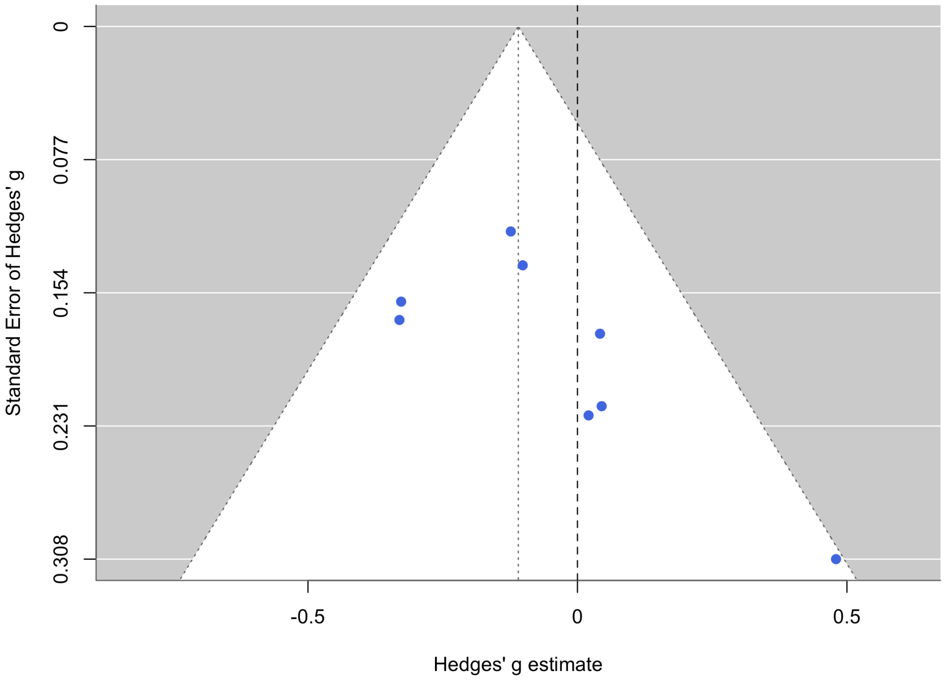

Figure 3 | Funnel plot for the secondary analysis of the EDE-Q subscale Shape Concern.

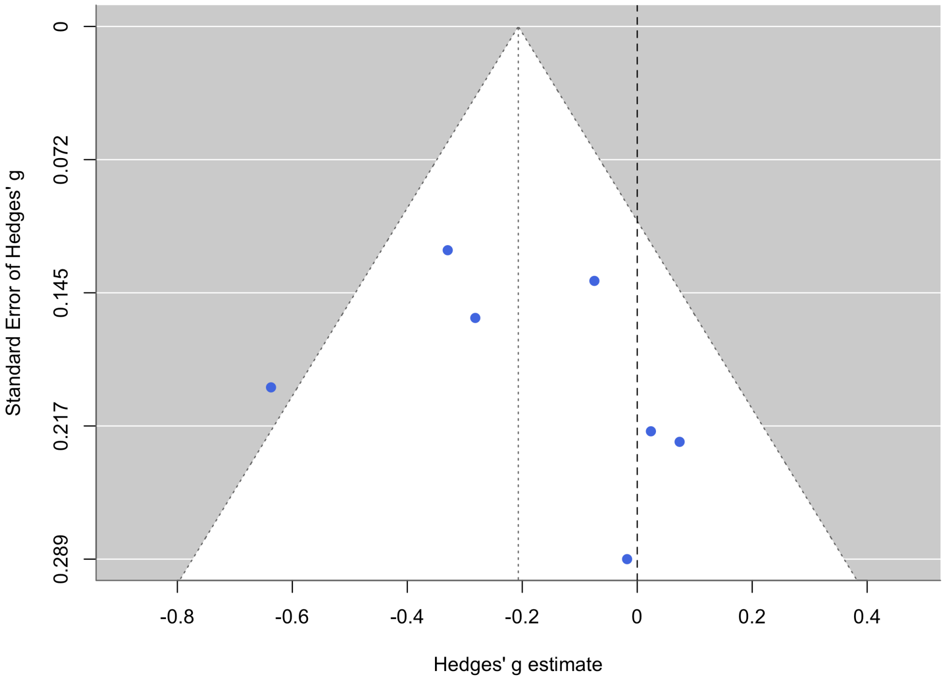

Figure 4 | Funnel plot for the secondary analysis of the EDE-Q subscale Eating Concern.

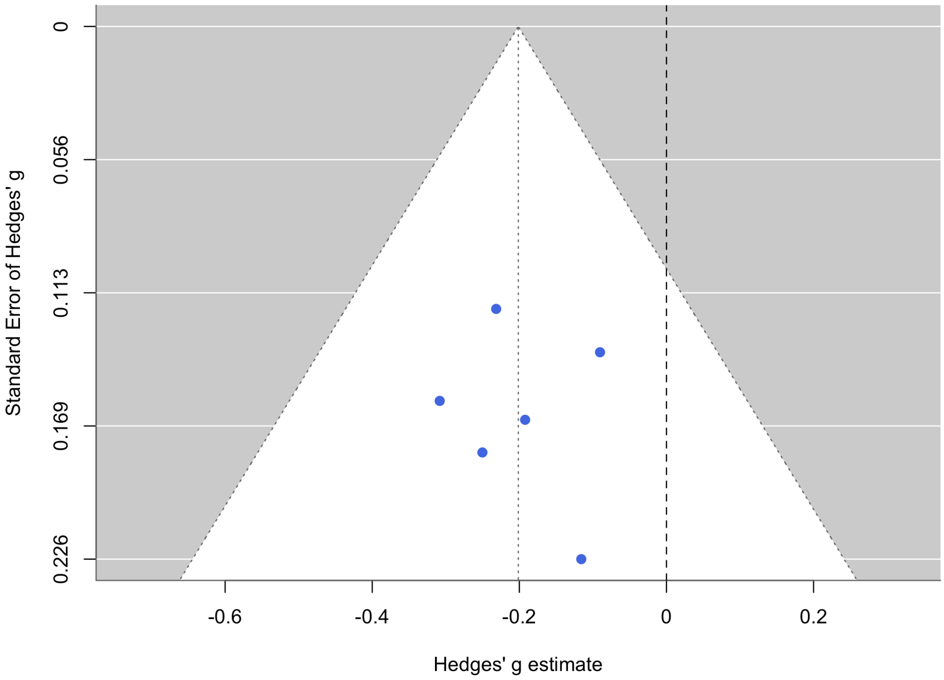

Figure 5 | Funnel plot for the sensitivity analysis only including RCTs.

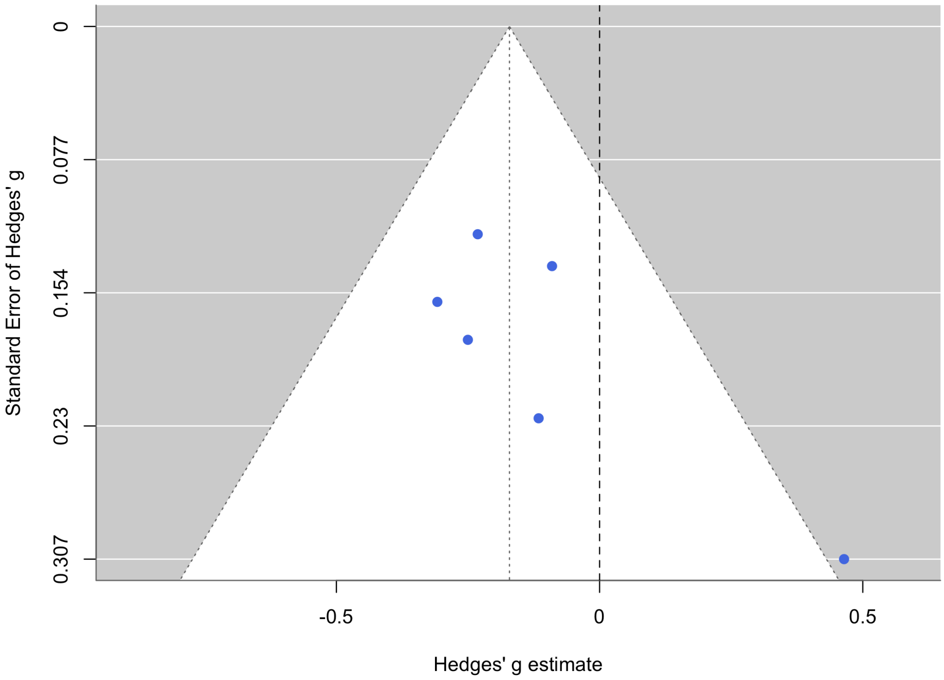

Figure 6 | Funnel plot for the sensitivity analysis excluding studies with differing WLCGs.

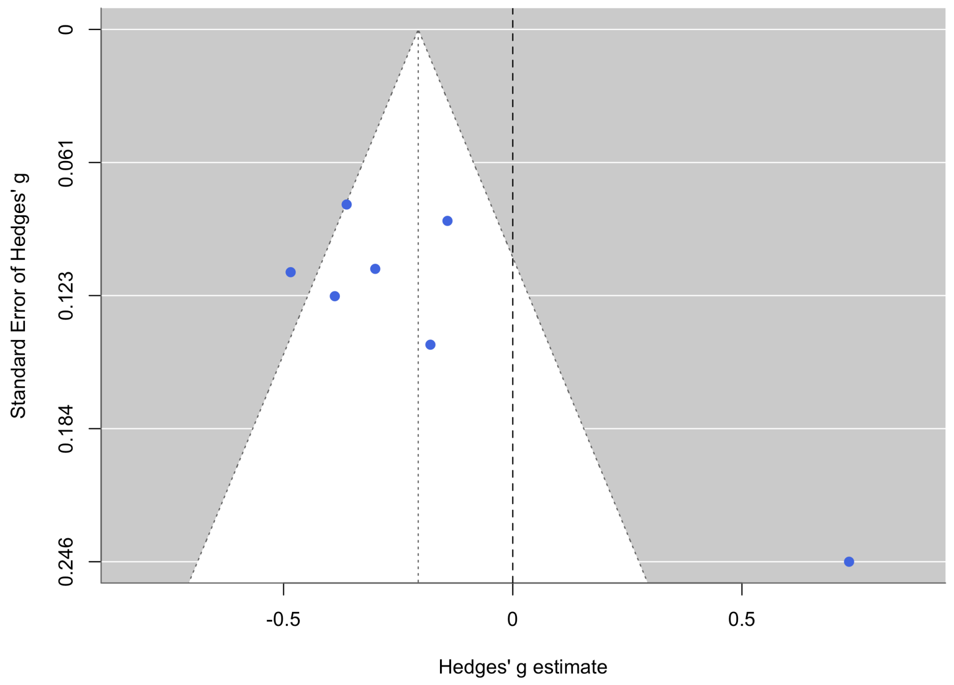

Figure 7 | Funnel plot for the sensitivity analysis assuming a pre-post correlation of r = 0.8.

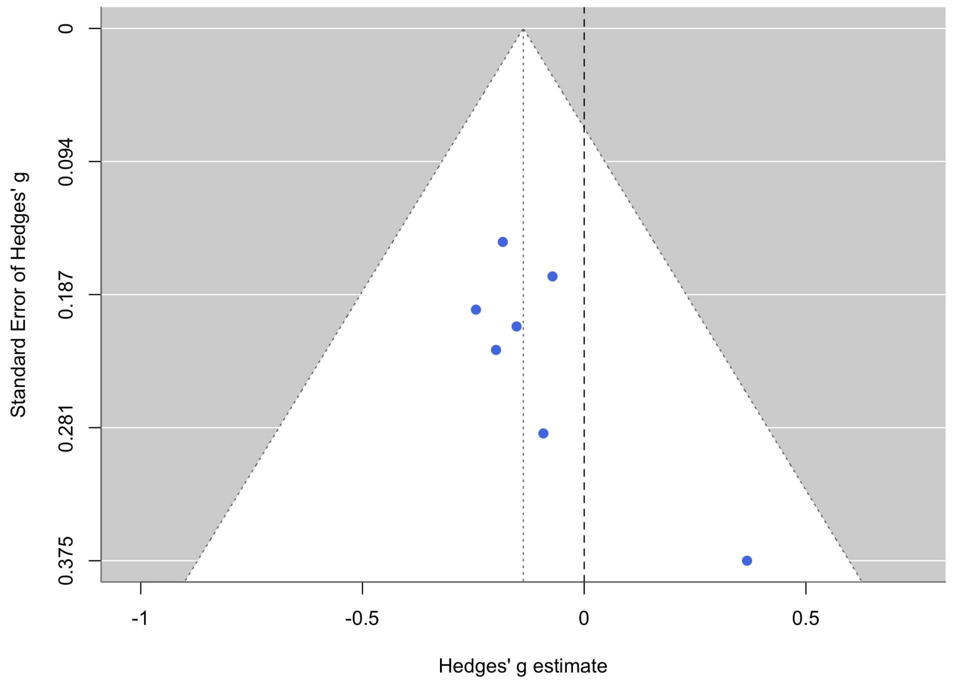

Figure 8 | Funnel plot for the sensitivity analysis assuming a pre-post correlation of r = 0.2.
