# Supplementary material for: The effects of waiting time for outpatient psychotherapeutic interventions on patient-reported outcomes in adolescents and adults with eating disorders: a systematic review and meta-analysis
Source: J Eat Disord. 2026 Jun 5;14:129. doi: 10.1186/s40337-026-01660-4 (PMC13248287; doi:10.1186/s40337-026-01660-4)
Supplement: Supplementary file 15 — Additional file 15. Decisions for each AMSTAR 2 item. [file 40337_2026_1660_MOESM15_ESM.pdf]

Additional file 15

Table | Decisions for each AMSTAR 2 item.

Domain	Item #	Item	Decision	Comment
Critical items	2	Did the report of the review contain an explicit statement that the review methods were established prior to the conduct of the review and did the report justify any significant deviations from the protocol?	Yes	A full protocol was pre-registered and the PROSPERO registration number was reported.
	4	Did the review authors use a comprehensive literature search strategy?	Yes	More than two databases were searched, search strings were provided, and no publication restrictions were applied.
	7	Did the review authors provide a list of excluded studies and justify the exclusions?	Yes	The reference list of excluded studies was provided at the end of the manuscript. Reasons for exclusions are provided in Figure 1.
	9	Did the review authors use a satisfactory technique for assessing the risk of bias in individual studies that were included in the review?	Yes	Risk of bias due to unconcealed allocation and lack of blinding of participants and assessors was assessed within the ROBINS-I and RoB 2 assessments.
	11	If meta-analysis was performed did the review authors use appropriate methods for statistical combination of results?	Yes	All statistical analyses were justified. An inverse variance weighting scheme was applied. Potential causes of heterogeneity were explored.
	13	Did the review authors account for risk of bias in individual studies when interpreting/discussing the results of the review?	Yes	The NRSI with a critical risk of bias was excluded in a sensitivity analysis. The reasons for high risk of bias across RCTs were reported and potential effects on the results were discussed.
	14	Did the review authors provide a satisfactory explanation for, and discussion of, any heterogeneity observed in the results of the review?	Yes	Statistical heterogeneity for the primary and secondary analyses was low.
Other items	1	Did the research questions and inclusion criteria for the review include the components of PICO?	Yes	We used a PECO scheme and predefined two mandatory time points.
	3	Did the review authors explain their selection of the study designs for inclusion in the review?	Yes	We provided an explanation for including both RCTs and NRSIs.
	5	Did the review authors perform study selection in duplicate?	Yes	Two authors performed study selection independently. If consensus could not be reached by discussion, a third author was consulted.

Domain	Item #	Item	Decision	Comment
	6	Did the review authors perform data extraction in duplicate?	Yes	Two authors performed data extraction independently. If consensus could not be reached by discussion, a third author was consulted.
	8	Did the review authors describe the included studies in adequate detail?	Yes	We described populations, exposures, comparators, outcomes and research designs. Detailed information on the populations and exposure was provided. Information on the comparator, i.e. the IG, was kept minimal due to the scope of our research question. Study settings and time frames for follow-up were described.
	10	Did the review authors report on the sources of funding for the studies included in the review? Note: Reporting that the reviewers looked for this information but it was not reported by study authors also qualifies.	Yes	We reported that sources of funding were reviewed with no notable concerns.
	12	If meta-analysis was performed, did the review authors assess the potential impact of risk of bias in individual studies on the results of the meta-analysis or other evidence synthesis?	Yes	All RCTs were judged to have a high risk of bias. The one NRSI had a critical risk and was excluded in a sensitivity analysis.
	15	If they performed quantitative synthesis did the review authors carry out an adequate investigation of publication bias (small study bias) and discuss its likely impact on the results of the review?	Yes	We visually inspected funnel plots and found no notable concerns regarding publication bias.
	16	Did the review authors report any potential sources of conflict of interest, including any funding they received for conducting the review?	Yes	We reported no competing interests and no funding for this systematic review and meta-analysis.

Note: AMSTAR = A MeaSurement Tool to Assess systematic Reviews[1]; IG = intervention group; NRSI = non-randomised study of intervention; PECO = population, exposure, comparator, outcome; RCT = randomised controlled trial; RoB 2 = Cochrane Risk of Bias 2; ROBINS-I = Risk of Bias in Non-randomised Studies of Interventions.

Reference List

1. Shea BJ, Reeves BC, Wells G, Thuku M, Hamel C, Moran J, et al. AMSTAR 2: a critical appraisal tool for systematic reviews that include randomised or non-randomised studies of healthcare interventions, or both. BMJ. BMJ; 2017;j4008. https://doi.org/10.1136/bmj.j4008
